# Clinical Outcomes of GLP-1 Receptor Agonist and SGLT2 Inhibitor Combination Therapy in Heart Failure: A Real-World Propensity-Matched TriNetX Analysis

**DOI:** 10.3390/biomedicines14061368

**Published:** 2026-06-17

**Authors:** Faizan Ahmed, Saifullah Khan, Najam Gohar, Muhammad Hassan, Madeeha Shafqat, Mushood Ahmed, Muhammad Hussain, Nisha Khalid, Mohammad Omar Butt, Haris Bin Tahir, Asma Naz, Tehmasp Rehman Mirza, Muhammad Abdullah, Abdul Hannan, Fenilkumar Kotadiya, Ameer Haider Cheema, Amro Taha, Fawaz Alenezi

**Affiliations:** 1 Department of Internal Medicine, Hackensack Meridian Health, Jersey Shore University Medical Center, Neptune, NJ 07753, USA; 2 Department of Internal Medicine, Dow University of Health & Sciences, Karachi 74200, Sindh, Pakistan; saifrah456@gmail.com (S.K.); azamh5223@gmail.com (M.H.); nishakhalid.2405@gmail.com (N.K.); asmynaz@gmail.com (A.N.); abdulhannanoffi@gmail.com (A.H.); 3 Department of Internal Medicine, Ameer-ud-Din Medical College, PGMI, Lahore 54000, Punjab, Pakistan; najamgoharr@gmail.com; 4 Department of Internal Medicine, Geisinger Medical Center, Danville, PA 17822, USA; drmadeehashafqat@gmail.com; 5 Department of Internal Medicine, Rawalpindi Medical University, Rawalpindi 46000, Punjab, Pakistan; mushood07@gmail.com; 6 Department of Dow Medical College, Dow University of Health & Sciences, Karachi 74200, Sindh, Pakistan; azamhhh7@gmail.com; 7 Department of Internal Medicine, Memorial Satilla Health, Waycross, GA 31501, USA; omar9224@gmail.com; 8 Department of Internal Medicine, Lahore General Hospital, Lahore 54000, Punjab, Pakistan; harristahirchh@gmail.com; 9 Department of Internal Medicine, Shalamar Medical and Dental College, Lahore 54000, Punjab, Pakistan; tehmaspmirza@gmail.com (T.R.M.); mohd_abdullah2003@outlook.com (M.A.); 10 Department of Internal Medicine, Charleston Area Medical Center, Charleston, WV 25301, USA; drfenilkotadiya@gmail.com; 11 Department of Internal Medicine, University of Texas Southwestern Medical Center, Dallas, TX 75390, USA; ameerhaider.cheema@utsouthwestern.edu; 12 Division of Cardiology, West Virginia University, Morgantown, WV 26506, USA; taha61591@gmail.com; 13 Division of Cardiology, Duke University Hospital, Durham, NC 27710, USA; fawaz.enezi@duke.edu

**Keywords:** heart failure, SGLT2 inhibitors, GLP-1 receptor agonists, combination therapy, propensity score matching

## Abstract

**Background**: Heart failure patients are frequently prescribed SGLT2 inhibitors, but the incremental real-world benefit of adding GLP-1 receptor agonists is uncertain. **Methods**: A retrospective propensity-matched cohort study was conducted using the TriNetX Global Collaborative Network (171 healthcare organizations). Adults aged ≥18 years with incident heart failure who initiated either dual SGLT2 inhibitor and GLP-1 receptor agonist therapy or SGLT2 inhibitor monotherapy within 1 month of the first heart failure diagnosis were compared. Outcomes over 365 days included all-cause mortality, all-cause hospitalization, acute myocardial infarction, atrial fibrillation/flutter, acute kidney failure, pulmonary edema, new-onset diuretic use, urinary tract infection, retinopathy, and laboratory hypoglycemia (glucose ≤ 70 mg/dL). Cox proportional hazards models were used; the proportional hazards assumption was formally tested. Bonferroni and Benjamini–Hochberg adjustments were applied for multiple comparisons. E-values quantified robustness to unmeasured confounding. Appendicitis was used as a negative control outcome. **Results**: After 1:1 propensity score matching, 3421 patients were included in each cohort. Cohorts were well-balanced (all standardized mean differences < 0.10). Over 365 days of follow-up, no significant differences were observed between dual therapy and monotherapy in all-cause mortality (HR 0.92, 95% CI 0.77–1.09), all-cause hospitalization (HR 1.16, 95% CI 0.96–1.40), acute myocardial infarction (HR 1.20, 95% CI 0.92–1.55), atrial fibrillation/flutter (HR 1.05, 95% CI 0.85–1.30), acute kidney failure (HR 1.05, 95% CI 0.89–1.26), new-onset diuretic use (HR 0.92, 95% CI 0.78–1.09), or urinary tract infection (HR 1.16, 95% CI 0.90–1.48). Dual therapy was associated with a significant increase in retinopathy (HR 2.66, 95% CI 1.81–3.93; Bonferroni *p* = 0.001; E-value 4.77, lower CI bound 3.01), and a modest increase in laboratory hypoglycemia (HR 1.22, 95% CI 1.04–1.44) and pulmonary edema (HR 1.35, 95% CI 1.06–1.70), both of which survived FDR but not Bonferroni adjustment. Prespecified subgroup analyses showed lower mortality with dual therapy in patients aged 18–64 (HR 0.64, 95% CI 0.43–0.93) and in women (HR 0.60, 95% CI 0.45–0.81). The negative control outcome (appendicitis) was non-significant. **Conclusions**: In a propensity-matched real-world cohort with a conservative cohort definition, dual SGLT2 inhibitor and GLP-1 receptor agonist therapy was not associated with significant differences in mortality or major cardiovascular outcomes compared with SGLT2 inhibitor monotherapy. Subgroup signals favoring dual therapy in younger and female patients, alongside safety signals for retinopathy and laboratory hypoglycemia, are hypothesis-generating and should be confirmed in prospective trials.

## 1. Introduction

Heart failure (HF) is a significant global health problem, with the estimates indicating that it impacts over 64 million people across the world and is also a significant contributor to morbidity, mortality, and health care expenditure. It is on the increase in tandem with the growing populations of the elderly and the escalating burden of cardiometabolic comorbidities such as type 2 diabetes mellitus (T2DM), obesity, and chronic kidney disease (CKD) [1]. HF has been linked to high hospitalization rates, diminished quality of life, and high economic costs, and thus, effective therapeutic interventions that not only alleviate symptoms but also reduce adverse clinical outcomes are required [2,3].

There has been a significant change in the management of HF over the last ten years as a result of new pharmacotherapies beyond conventional neurohormonal blockade [4]. Sodium-glucose co-transporter-2 (SGLT2) inhibitors have shown strong improvements across the entire continuum of HF (hospitalization and cardiovascular mortality), regardless of diabetes status [5,6]. At the same time, the glucagon-like peptide-1 receptor agonists (GLP-1 RAs) have demonstrated positive outcomes in terms of weight loss, glycemic regulation, and atherosclerotic cardiovascular results, and there is emerging evidence that they can positively impact HF-related pathways by regulating inflammation, myocardial metabolism, and endothelial function [7,8].

The combination therapy using GLP-1 RAs and SGLT2 inhibitors is a potential therapy option in patients with HF, and especially in those with co-morbid metabolic disease. The main effects of SGLT2 inhibitors are hemodynamic and renal protective effects, whereas GLP-1 RAs enhance metabolic profiles and can mitigate systemic inflammation and vascular dysfunction. These agents, when combined, can have synergistic effects by addressing multiple pathophysiological mechanisms that contribute to HF [9].

Although this rationale is quite strong in a mechanistic context and there is increasing clinical interest in this approach, there is still limited large-scale real-world evidence to assess the effectiveness and safety of combined GLP-1 RA and SGLT2 inhibitor therapy in HF patients. The majority of randomized controlled trials have compared these agents as single agents rather than in combination, and the available observational studies are usually limited by small sample sizes, non-homogeneous populations, and inadequate adjustment for baseline differences [10,11]. Also, relative results on patients undergoing combination therapy in comparison to monotherapy or standard care, especially in various real-life populations, have not been well established.

Thus, with the TriNetX global federated research network, we performed a large-scale real-world propensity score-matched study to assess the clinical outcome of GLP-1 receptor agonist and SGLT2 inhibitor combination therapy in patients with heart failure. We paired patients who underwent combination therapy with suitable comparator groups with major adverse cardiovascular events, heart failure hospitalization, all-cause mortality, and other clinically important outcomes in short and long-term follow-up.

## 2. Methods

### 2.1. Data Source

This retrospective cohort was conducted using the TriNetX Research Network (TriNetX LLC, Cambridge, MA, USA), a global federated network that provides access to de-identified electronic medical records, including diagnoses, procedures, medications, laboratory values, and genomic information, across large healthcare organizations (HCOs). The query for this data analysis was generated on 20 May 2026, drawing data from 171 HCOs within the Global Collaborative Network. TriNetX aggregates patient-level clinical data and complies with the Health Insurance Portability and Accountability Act (HIPAA) (Supplemental Table S1). As this retrospective cohort study used the TriNetX research network, which provides access to de-identified and aggregated electronic health record data from participating healthcare organizations, no directly identifiable patient information was accessed. In accordance with TriNetX data use policies and applicable institutional guidelines, studies using such de-identified datasets are generally considered exempt from institutional review board (IRB) oversight and informed consent requirements. However, ethical requirements may vary across institutions, and the study was conducted in compliance with relevant data protection and ethical standards, including principles of patient confidentiality and good clinical practices.

### 2.2. Study Population and Design

**Figure 1:**
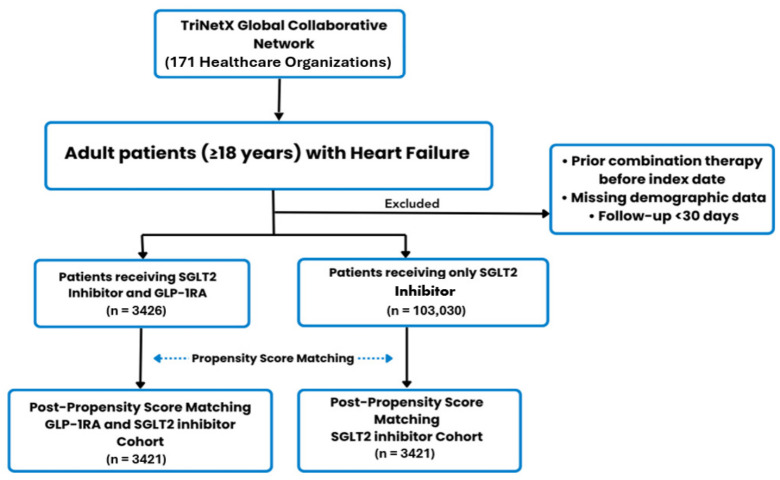
Study Flow Diagram.

The study included patients aged 18 years or older. Patients were categorized into two groups: the DUAL group (Cohort A, *n* = 9734) and the Mono group (Cohort B, *n* = 128,606) before propensity score matching (PSM). Both cohorts were required to have a diagnosis of heart failure. The DUAL cohort was defined by the concurrent use of sodium-glucose co-transporter 2 (SGLT2) inhibitors and glucagon-like peptide-1 (GLP-1) analogues (Supplemental Table S1). The Mono cohort was defined by the use of SGLT2 inhibitors and the absence of GLP-1 analogues (Supplemental Table S2). The index event was defined as the date of the first instance of medication exposure (SGLT2 inhibitors with or without GLP-1 analogues), provided that this record occurred within a 1-year window on or after the initial diagnosis of heart failure. To reduce immortal time bias and confounding by treatment intensity, cohorts were restricted to patients initiating qualifying therapy within 1 month after the first heart failure diagnosis. Exposure reflected prescription overlap recorded in TriNetX and did not capture medication adherence, persistence, switching, or daily dose. Patients who did not meet this temporal criterion were excluded. To ensure data relevance, only index events occurring within the last 20 years were included (Figure 1). A 1-year baseline lookback period prior to the index date was applied to capture covariates. Exposure definition required documented concurrent prescription overlap between SGLT2 inhibitors and GLP-1 receptor agonists during the index period. Dual therapy was defined as overlapping use of both drug classes within the TriNetX exposure window. Exposure to SGLT2 inhibitors and GLP-1 receptor agonists was defined at the class level using prescription records within the network. SGLT2 inhibitors (ATC A10BK) included empagliflozin, dapagliflozin, canagliflozin, ertugliflozin, sotagliflozin, bexagliflozin, ipragliflozin, tofogliflozin, luseogliflozin, remogliflozin, and henagliflozin. GLP-1 receptor agonists (ATC A10BJ) included exenatide, liraglutide, lixisenatide, albiglutide, dulaglutide, semaglutide, beinaglutide, and tirzepatide (dual GIP/GLP-1 receptor agonist).

### 2.3. Study Endpoints

Clinical outcomes were assessed over a 365-day follow-up period starting 1 day after the index event. The outcomes included mortality, acute myocardial infarction (AMI), atrial fibrillation and flutter (AFIB), and acute kidney (AK) failure. Additional outcomes assessed were pulmonary edema (PE), new-onset diuretic use, urinary tract infection (UTI), retinopathy, and hypoglycemia (Supplemental Table S3).

New-onset diuretic use was defined as the first prescription of any ATC C03 diuretic (loop, thiazide, or potassium-sparing) during the 365-day follow-up among patients without prior diuretic prescription in the baseline period.

Mortality was identified by “Deceased” status in demographic records. AMI was defined as an acute myocardial infarction. AFIB was defined as atrial fibrillation and flutter. Acute kidney failure was defined using relevant diagnostic codes. Retinopathy was defined as a composite outcome including various type 2 diabetes mellitus-related retinal disorders and ophthalmic complications. All outcomes were identified using ICD-10-CM coded diagnoses without manual chart validation, as is inherent to the use of de-identified EHR-based databases (Supplemental Table S4).

Outcomes were identified using ICD-10 coding without manual chart validation. In addition to relative measures (hazard ratios and confidence intervals), absolute risk differences and number needed to treat or harm (NNT/NNH) were calculated for outcomes retaining statistical significance to improve assessment of clinical magnitude. All-cause hospitalization was additionally evaluated as a clinically relevant outcome. Outcome ascertainment relied on ICD-10 coding within TriNetX and should be interpreted with recognition of potential coding-related misclassification.

### 2.4. Statistical Analysis

To mitigate baseline differences between the cohorts, PSM was conducted. From the initial pool, 1:1 matching was performed, resulting in balanced cohorts of 9731 patients each [12,13] (Supplementary Figure S1). Matching criteria included demographic factors (age, sex, and ethnicity), comorbid conditions (diabetes mellitus, obesity, and malnutrition), procedures (e.g., cardiovascular investigations), and medications (including ACE inhibitors, beta blockers, and diuretics).

**Figure 2:**
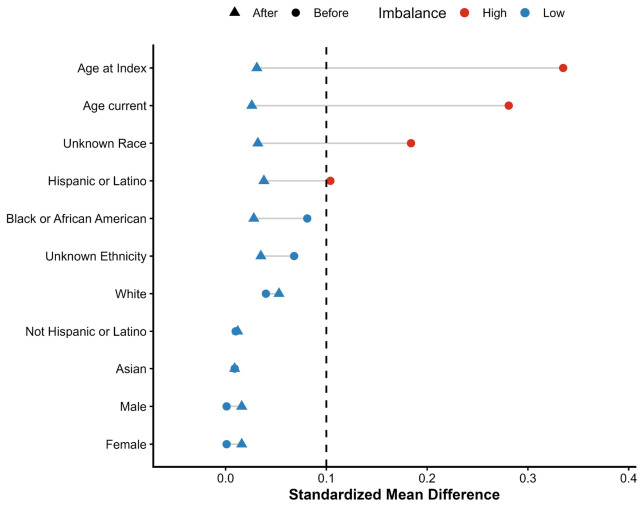
Love Plot Demonstrating Balance After Propensity Score Matching For Demographics.

**Figure 3:**
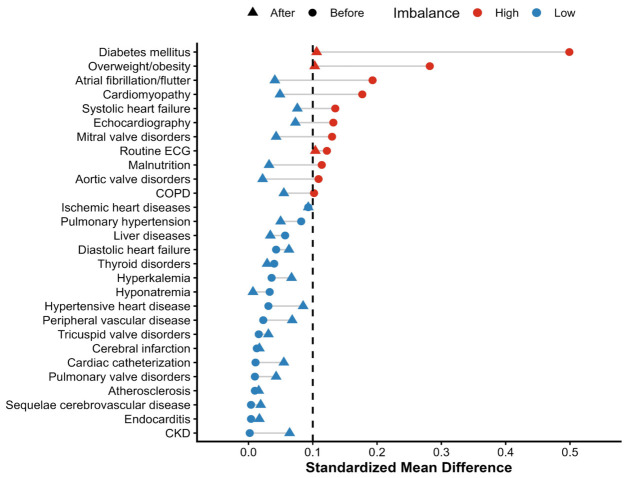
Love Plot Demonstrating Balance After Propensity Score Matching For Comorbidities.

**Figure 4:**
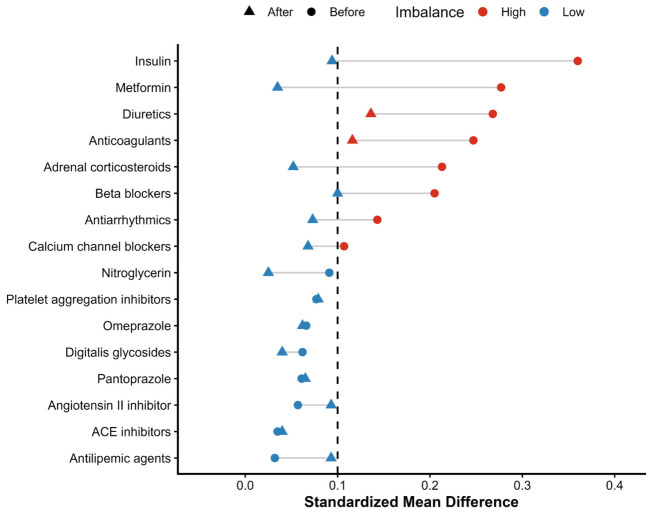
Love Plot Demonstrating Balance After Propensity Score Matching For Medications.

**Figure 5:**
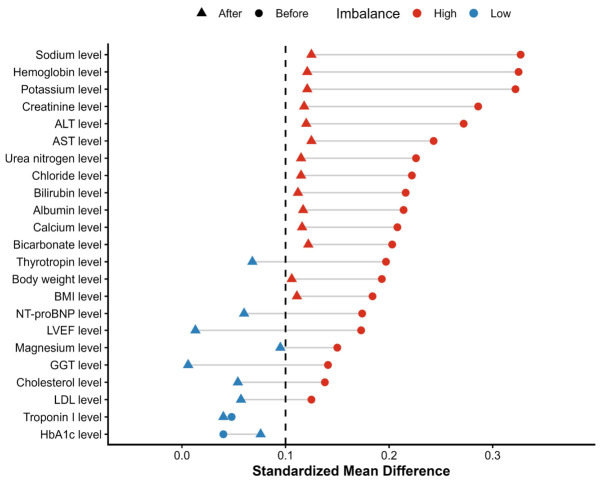
Love Plot Demonstrating Balance After Propensity Score Matching For Laboratories.

Propensity scores were estimated using logistic regression within the TriNetX platform. Nearest-neighbor 1:1 matching without replacement was applied using a caliper of 0.1 standard deviations of the logit of the propensity score. Covariate balance was assessed using standardized mean differences (SMD), with SMD < 0.1 indicating adequate balance. Covariate balance was visually assessed using Love plots (Figure 2, Figure 3, Figure 4 and Figure 5). Propensity score matching was performed using 1:1 nearest-neighbor matching without replacement. Under this approach, the final matched cohort size is constrained by the smaller of the two pre-match treatment groups. Consequently, unmatched patients from the larger SGLT2 inhibitor monotherapy cohort were excluded when no acceptable propensity score counterpart existed within the predefined caliper. This reflects the expected behavior of propensity score matching and represents the selection of the most clinically and demographically comparable control patients rather than the loss of information from the dual-therapy cohort.

Incidence risks and measures of association, specifically Risk Ratios (RRs) and Odds Ratios (ORs) with 95% confidence intervals (CIs), were calculated using the TriNetX Compare Outcomes model. Time-to-event data were visualized using Kaplan–Meier survival curves. Hazard ratios (HRs) and 95% CIs were derived from Cox proportional hazards regression models within the platform [14]. A two-sided *p*-value of less than 0.05 was considered statistically significant.

The proportional hazards assumption was assessed within the TriNetX platform framework. No missing data imputation was performed, and analyses were based on available electronic health record data only. No formal sensitivity analyses were conducted.

Prespecified sensitivity analyses were conducted according to age group, sex, and heart failure phenotype. Quantitative bias analyses using E-values were performed for statistically significant associations. Appendicitis was assessed as a negative control outcome. To account for multiple outcome testing, both Bonferroni-adjusted *p*-values and Benjamini–Hochberg false discovery rate (BH-FDR) q-values were calculated across the ten predefined outcomes. Statistical significance after Bonferroni correction was defined as *p* < 0.005. The proportional hazards assumption for Cox regression analyses was formally tested using the TriNetX proportionality test. Absolute risk differences and numbers needed to treat or harm (NNT/NNH) were additionally calculated for statistically significant outcomes to facilitate assessment of clinical relevance.

## 3. Results

### 3.1. Patient Characteristics

The initial study population comprised 3426 patients receiving dual SGLT2 inhibitor and GLP-1 analogue therapy and 103,030 patients receiving SGLT2 inhibitor monotherapy (Table 1). Given the substantially larger size of the SGLT2 inhibitor monotherapy cohort, 1:1 propensity-score matching was performed to generate balanced and comparable study groups. The substantial reduction in the SGLT2 inhibitor monotherapy cohort after matching reflects the standard mechanics of 1:1 nearest-neighbor propensity-score matching without replacement, whereby the matched control cohort was constrained to the size of the smaller exposure cohort after caliper restrictions. The resulting matched controls represented the subset of monotherapy patients with sufficient overlap in baseline covariates to serve as appropriate comparators, whereas unmatched patients were excluded because their baseline characteristics fell outside the region of common support, where causal inference is least reliable. After matching, the two groups were well balanced, with 3421 patients retained in each cohort. Baseline demographic and clinical characteristics before and after matching are summarized in Table 1.

Before propensity-score matching, the mean age at index was 62.6 ± 12.4 years and 67.0 ± 14.0 years, respectively (SMD = 0.335). 63.5% of both the dual therapy and monotherapy cohorts were males (SMD = 0.001). Per race and ethnicity, White patients made 56.4% of the dual therapy cohort and 54.4% of the monotherapy cohort (SMD = 0.040), whereas Black or African American patients represented 19.6% and 16.4%, respectively (SMD = 0.081). Hispanic or Latino patients were 7.8% of the dual therapy cohort and 5.2% of the monotherapy cohort (SMD = 0.104). For baseline comorbidities, diabetes mellitus was prevalent in 59.1% of the dual therapy group and 34.9% of the monotherapy group (SMD = 0.499), whereas overweight, obesity, and other hyperalimentation disorders were recorded in 31.9% and 19.7%, respectively (SMD = 0.282). Ischemic heart disease was a burden in 43.5% of the dual therapy cohort and 48.2% of the monotherapy cohort (SMD = 0.093), while systolic congestive heart failure was observed in 34.6% and 41.1%, respectively (SMD = 0.135). Regarding procedures, echocardiography procedures were documented in 32.2% of the dual therapy cohort and 38.5% of the monotherapy cohort (SMD = 0.132), while routine electrocardiography with at least 12 leads was recorded in 43.3% and 49.4%, respectively (SMD = 0.122). Regarding medication use, diuretic use was documented in 45.4% of the dual therapy cohort and 58.6% of the monotherapy cohort (SMD = 0.268). Insulin use was observed in 45.9% of the dual therapy group and 28.8% of the monotherapy group (SMD = 0.360), whereas metformin use was reported in 17.3% and 8.2%, respectively (SMD = 0.277). Baseline laboratory and anthropometric variables included mean hemoglobin levels of 12.4 ± 2.5 g/dL and 12.3 ± 2.6 g/dL, respectively (SMD = 0.325). Mean hemoglobin A1c was 8.5 ± 2.4% in the dual therapy cohort and 6.7 ± 1.9% in the monotherapy cohort (SMD = 0.040). Mean body mass index was recorded as 35.0 ± 9.3 kg/m^2^ and 30.1 ± 8.1 kg/m^2^, respectively (SMD = 0.184), while mean body weight was 223.8 ± 67.5 lb in the dual therapy cohort and 192.8 ± 57.4 lb in the monotherapy cohort (SMD = 0.193). Mean left ventricular ejection fraction was observed as 43.3 ± 16.4% in the dual therapy group and 38.9 ± 16.2% in the monotherapy group (SMD = 0.173).

Following propensity-score matching, the mean age at index was 62.6 ± 12.4 years and 63.0 ± 13.5 years, respectively (SMD = 0.031). Male patients accounted for 63.5% and 62.7% of the matched cohorts (SMD = 0.016). White patients comprised 56.4% of the dual therapy cohort and 59.0% of the monotherapy cohort (SMD = 0.053), while Black or African American patients represented 19.6% and 18.5%, respectively (SMD = 0.028). Hispanic or Latino patients represented 7.8% and 6.8%, respectively (SMD = 0.038).

After matching, diabetes mellitus was recorded in 59.0% of the dual therapy cohort and 53.8% of the monotherapy cohort (SMD = 0.106), while overweight, obesity, and other hyperalimentation disorders were present in 31.9% and 27.2%, respectively (SMD = 0.103). Ischemic heart disease was documented in 43.5% and 39.0% of patients, respectively (SMD = 0.093), while systolic congestive heart failure was present in 34.6% and 31.0% (SMD = 0.076). Regarding procedures, echocardiography procedures were documented in 32.2% and 28.8% of the matched cohorts (SMD = 0.073), while routine electrocardiography with at least 12 leads was reported in 43.3% and 38.2%, respectively (SMD = 0.104). Diuretic use was reported in 45.4% of the dual therapy cohort and 38.7% of the monotherapy cohort (SMD = 0.136). Insulin use was observed in 45.8% and 41.2%, respectively (SMD = 0.094), whereas metformin use was reported in 17.3% and 16.0% (SMD = 0.035). Among laboratory and anthropometric variables after matching, the mean hemoglobin levels were 12.4 ± 2.6 g/dL and 12.4 ± 2.7 g/dL, respectively (SMD = 0.121). Mean hemoglobin A1c was 8.5 ± 2.4% in the dual therapy cohort and 7.6 ± 2.2% in the monotherapy cohort (SMD = 0.076). Mean body mass index was 35.0 ± 9.3 kg/m^2^ and 33.0 ± 8.6 kg/m^2^, respectively (SMD = 0.111), while mean body weight was 223.8 ± 67.5 lb and 210.4 ± 59.2 lb, respectively (SMD = 0.106). Mean left ventricular ejection fraction was 43.3 ± 16.4% in the dual therapy cohort and 41.7 ± 16.8% in the monotherapy cohort (SMD = 0.013). (Supplementary Figure S2).

### 3.2. Outcomes

#### 3.2.1. All-Cause Mortality

**Figure 6:**
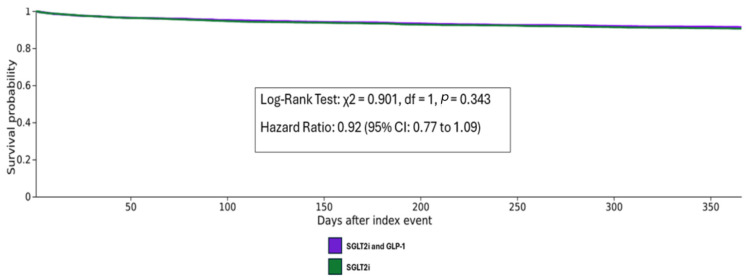
Kaplan–Meier curves for All-Cause Mortality at 1 Year Follow-up.

At 1 year, all-cause mortality was recorded in 7.35% of those receiving SGLT2 inhibitor plus GLP-1 analogue dual therapy, compared with 7.84% treated with SGLT2 inhibitor monotherapy. Kaplan–Meier survival analysis showed an HR of 0.92 (95% CI: 0.77 to 1.09; log-rank *p*: 0.343; Bonferroni-adjusted *p*: 1; BH-FDR q: 0.443), indicating a non-significant lower hazard in the dual therapy cohort. Furthermore, the PH assumption for this outcome was satisfied. Overall, these findings should be interpreted with caution, as they are hypothesis-generating and may be influenced by residual confounding (Table 2) (Figure 6) (Supplementary Tables S8 and S10).

#### 3.2.2. All-Cause Hospitalization

Over 365 days of follow-up, no statistically significant difference was observed in all-cause hospitalization between dual therapy and SGLT2 inhibitor monotherapy cohorts (HR: 1.16, 95% CI: 0.96 to 1.4; log-rank *p*: 0.134; Bonferroni-adjusted *p*: 1; BH-FDR q: 0.335). The PH test was satisfactory for this finding. Findings remained non-significant following multiple-comparison adjustment. Because heart failure-specific hospitalization could not be reliably isolated within the de-identified TriNetX dataset, all-cause hospitalization was used as the clinically relevant hospitalization endpoint (Table 2) (Supplementary Figure S2, Tables S8 and S10).

#### 3.2.3. Acute Myocardial Infarction

At the 1-year follow-up, acute myocardial infarction events were documented in 4.79% of patients in the dual therapy group and 3.96% patients in the monotherapy group. Time-to-event analysis using Kaplan–Meier methods produced an HR of 1.2 (95% CI: 0.92 to 1.55; log-rank *p*: 0.176; Bonferroni-adjusted *p*: 1; BH-FDR q: 0.352), demonstrating a non-significant, slightly increased risk with dual therapy. Additionally, this outcome did not satisfy the PH test (Table 2) (Supplementary Figure S3, Tables S8 and S10).

#### 3.2.4. Atrial Fibrillation and Flutter

Over 1 year of follow-up, atrial fibrillation or flutter developed in 7.18% of patients assigned to dual therapy and 6.65% of patients receiving monotherapy. Kaplan–Meier estimates showed an HR of 1.05 (95% CI: 0.85 to 1.3; log-rank *p*: 0.65; Bonferroni-adjusted *p*: 1; BH-FDR q: 0.65), suggesting a non-significant difference with a slightly increased hazard in the dual therapy cohort. Furthermore, this outcome satisfied the PH assumption (Table 2) (Supplementary Figure S4, Tables S8 and S10).

#### 3.2.5. Acute Kidney Failure

At 1 year, acute kidney failure occurred in 10.48% of the dual therapy cohort versus 9.67% of the monotherapy cohort. Kaplan–Meier analysis revealed an HR of 1.05 (95% CI: 0.89 to 1.26; log-rank *p*: 0.554; Bonferroni-adjusted *p*: 1; BH-FDR q: 0.616), demonstrating a non-significantly elevated hazard in patients receiving dual therapy. Moreover, the PH assumption for this outcome was satisfied (Table 2) (Supplementary Figure S5, Tables S8 and S10).

#### 3.2.6. Pulmonary Edema

During a 1-year follow-up, pulmonary edema was identified in 5.29% treated with dual therapy and 3.88% treated with monotherapy. Kaplan–Meier analysis yielded an HR of 1.35 (95% CI: 1.06 to 1.7; log-rank *p*: 0.013; Bonferroni-adjusted *p*: 0.14; BH-FDR q: 0.05), showing a significantly increased risk in the dual therapy cohort. The absolute increase in risk was 1.4%, corresponding to an NNH of 71. Additionally, this outcome satisfied the PH assumption (Table 2) (Supplementary Figure S6, Tables S8, S10 and S11).

The sensitivity analysis performed resulted in an E-value of 2.03 (Lower CI: 1.32) for the outcome (Supplementary Table S6).

#### 3.2.7. New Onset Diuretic Use

At 1 year, initiation of diuretic therapy was observed in 37.56% of the dual therapy group compared with 39.34% of the monotherapy group. Kaplan–Meier estimates showed an HR of 0.92 (95% CI: 0.78 to 1.09; log-rank *p*: 0.354; Bonferroni-adjusted *p*: 1; BH-FDR q: 0.443), suggesting a non-significant lower hazard among those receiving dual therapy. The outcome did not satisfy the PH test (Table 2) (Supplementary Figure S7, Tables S8 and S10).

#### 3.2.8. Urinary Tract Infection

Urinary tract infection occurred in 4.35% patients in the dual therapy cohort and 3.68% patients in the monotherapy cohort at the 1-year follow-up. Kaplan–Meier analysis yielded an HR of 1.16 (95% CI: 0.9 to 1.48; log-rank *p*: 0.253; Bonferroni-adjusted *p*: 1; BH-FDR q: 0.417), indicating a statistically non-significant difference, although the absolute difference between groups was small. Moreover, the PH assumption for this outcome was satisfied (Table 2) (Supplementary Figure S8, Tables S8 and S10).

#### 3.2.9. Retinopathy

At the 1-year follow-up, retinopathy was reported in 2.88% of those receiving dual therapy and 1.06% of those receiving monotherapy. In the revised conservative analysis designed to reduce immortal time bias and treatment-intensity confounding, dual therapy remained associated with an increased hazard of retinopathy (HR: 2.66, 95% CI: 1.81 to 3.93; log-rank *p*: 0; Bonferroni-adjusted *p*: 0.001; BH-FDR q: 0.001). The absolute risk increase was 1.8%, corresponding to a number needed to harm (NNH) of 56. The PH test for this outcome was also satisfied. Given the observational design, residual confounding, differences in diabetes severity, and surveillance bias may have contributed to this finding; therefore, interpretation should remain cautious. (Table 2) (Supplementary Figure S9, Tables S8, S10 and S11).

Sensitivity analysis for quantitative bias had an E-value of 4.77 (Lower CI: 3.01) for the outcome (Supplementary Table S9).

#### 3.2.10. Hypoglycemia

Over 1 year, hypoglycemia was observed in 10.27% of patients in the dual therapy cohort and 8.33% of patients in the monotherapy cohort. Kaplan–Meier analysis showed an HR of 1.22 (95% CI: 1.04 to 1.44; log-rank *p*: 0.015; Bonferroni-adjusted *p*: 0.15; BH-FDR q: 0.05), suggesting a statistically significant higher hazard with dual therapy. The absolute risk increase was 2.0%, corresponding to an NNH of 50. Additionally, the outcome satisfied the PH test (Table 2) (Supplementary Figure S10, Tables S8, S10 and S11).

Sensitivity analysis calculated an E-value of 1.75 (Lower CI: 1.24) for the outcome (Supplementary Table S9).

### 3.3. Heart Failure Subgroup Analyses

#### 3.3.1. HFrEF Subgroup

In the HFrEF subgroup (2015 matched patients in each cohort), the overall findings were largely consistent with the principal analysis. All-cause mortality was similar between groups (HR 0.98, 95% CI 0.79 to 1.22), consistent with the primary analysis. Most other outcomes, including acute myocardial infarction (HR 1.01), atrial fibrillation/flutter (HR 1.03), acute kidney injury (HR 1.04), new onset diuretic use (HR 1.09), and hypoglycemia (HR 1.01), also showed non-significant differences in line with the principal analysis. However, all-cause hospitalization was significantly higher with dual therapy (HR 1.49, 95% CI 1.13 to 1.97), representing a divergence from the principal analysis. Retinopathy was significantly increased with dual therapy (HR 1.87, 95% CI 1.15 to 3.03), consistent in direction with the principal analysis. While pulmonary edema showed a non-significantly higher risk (HR 1.25, 95% CI 0.95 to 1.65), it differed from the primary analysis. The complete outcome estimates for the HFrEF subgroup are provided in Supplementary Table S4.

#### 3.3.2. HFpEF Subgroup

In the HFpEF subgroup (1380 matched patients in each cohort), the pattern was broadly consistent with the principal analysis, although some associations were stronger in magnitude. All-cause mortality showed no significant difference between the two groups (HR 1.17, 95% CI 0.90 to 1.52), consistent with the principal analysis. All-cause hospitalization was substantially higher with the HFpEF subgroup (HR 1.79, 95% CI 1.20 to 2.68). Similarly, pulmonary edema (HR 1.52, 95% CI 1.03 to 2.24), retinopathy (HR 1.96, 95% CI 1.13 to 3.40) and hypoglycemia (HR 1.28, 95% CI 0.98 to 1.66) were increased with dual therapy, consistent with the principal findings. Other outcomes, including acute myocardial infarction (HR 1.06), atrial fibrillation/flutter (HR 1.13), acute kidney injury (HR 1.14), new onset diuretic use (HR 0.98), and urinary tract infection (HR 1.03), were largely similar to the principal analysis. Detailed results for the HFpEF subgroup are presented in Supplementary Table S5.

### 3.4. Age Subgroup Analyses

In both pre-specified age groups, most outcomes showed a similar direction of effect as the primary analysis, although effect sizes and statistical significance varied. In patients aged 18–64 years, all-cause mortality was significantly lower with the dual therapy group (HR 0.64, 95% CI 0.43 to 0.93), showing a stronger benefit than the principal analysis. In patients aged ≥65 years, all-cause mortality was neutral (HR 1.00, 95% CI 0.81 to 1.22), consistent with the principal analysis. Retinopathy risk was significantly higher with dual therapy in both younger (HR 2.29, 95% CI 1.27 to 4.11) and older patients (HR 2.46, 95% CI 1.50 to 4.03), in line with the principal findings. All-cause hospitalization showed a non-significant increase in both age groups (HR 1.36 and 1.22, respectively). The pattern for other outcomes including acute myocardial infarction, atrial fibrillation/flutter, acute kidney injury, pulmonary edema, new onset diuretic use, urinary tract infection, and hypoglycemia was broadly consistent with the principal analysis. Outcome estimates for both age strata are provided in Supplementary Table S6.

### 3.5. Gender Subgroup Analyses

Across gender strata, findings were mostly concordant with the principal analysis. Among males, all-cause mortality was non-significantly lower with dual therapy (HR 0.92, 95% CI 0.76 to 1.13), aligning closely with the principal analysis. In contrast, females showed a significant reduction in mortality with dual therapy (HR 0.60, 95% CI 0.45 to 0.81). All-cause hospitalization was not significantly different in males (HR 1.09, 95% CI 0.86 to 1.39) but was significantly increased in females (HR 1.57, 95% CI 1.13 to 2.18). Whereas retinopathy remained significantly higher in both males (HR 2.20, 95% CI 1.41 to 3.44) and females (HR 2.70, 95% CI 1.46 to 4.96), consistent with the principal analysis. Other outcomes including acute myocardial infarction, atrial fibrillation/flutter, acute kidney injury, pulmonary edema, new onset diuretic use, urinary tract infection, and hypoglycemia were broadly similar in direction to the overall cohort. Outcome estimates for both gender strata are provided in Supplementary Table S7.

### 3.6. Multiple Comparison Adjustment and Proportional Hazards Testing

After adjustment for multiple comparisons, only retinopathy remained statistically significant following Bonferroni correction (adjusted *p* = 0.001). Hypoglycemia and pulmonary edema retained borderline significance under BH-FDR adjustment (q = 0.05) but did not remain significant following Bonferroni correction. All other outcomes were non-significant after either adjustment (Supplementary Table S8).

The proportional hazards assumption was formally tested for all outcomes using the TriNetX implementation. The assumption held for 8 of 10 outcomes (*p* > 0.05) (Supplemental Table S10). Violations were observed for new-onset diuretic use (*p* = 0.030) and acute myocardial infarction (*p* = 0.008), indicating that corresponding HRs should be interpreted as weighted averages of potentially time-varying effects.

## 4. Discussion

**Figure 7:**
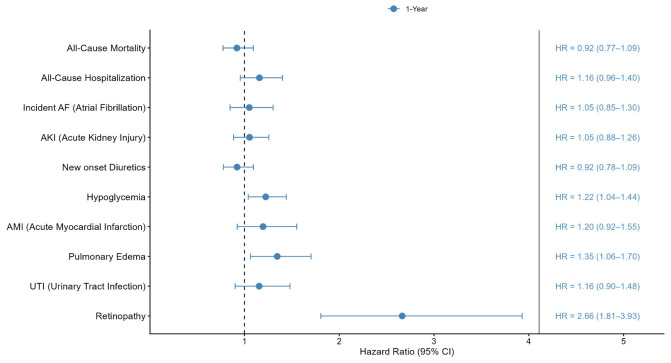
Forest plot of clinical outcomes comparing SGLT2 inhibitor + GLP-1 analogue dual therapy against SGLT2 inhibitor monotherapy in heart failure patients.

In this large propensity score-matched cohort of patients with heart failure, dual therapy with SGLT2 inhibitors and GLP-1 analogues was not associated with significant differences in all-cause mortality, acute myocardial infarction, acute kidney failure, atrial fibrillation/flutter, all-cause hospitalization, or new-onset diuretic use compared with SGLT2 inhibitor monotherapy over 365 days of follow-up, while pulmonary edema and hypoglycemia showed borderline significant increases. The absence of significant differences across several aforementioned endpoints strengthens the overall consistency of the study findings. Rather than being limited to a single outcome, the neutral associations were observed across multiple domains, reducing the likelihood that the primary results were driven by isolated endpoint-specific variation. Baseline demographic, clinical, and laboratory characteristics were well balanced after matching, suggesting adequate covariate balance and highlighting the robustness of the matching procedure. While the overall cohort did not demonstrate a mortality benefit, subgroup analyses demonstrated lower mortality among younger patients and women receiving dual therapy, suggesting that the effect of combination therapy may differ among specific patient populations. Additionally, retinopathy, laboratory-defined hypoglycemia, and pulmonary edema emerged as potential safety signals. (Figure 7) (Graphical Abstract). However, given the observational design, these associations should be interpreted as hypothesis-generating rather than causal and may be influenced by residual confounding, including confounding by indication and treatment selection bias.

Current guidelines recommend distinct pharmacological strategies across heart failure subtypes defined by left ventricular ejection fraction. In patients with heart failure with reduced ejection fraction (HFrEF), guideline-directed medical therapy includes renin–angiotensin–aldosterone system inhibitors, beta-blockers, mineralocorticoid receptor antagonists, and SGLT2 inhibitors, all of which have demonstrated mortality benefit. In heart failure with mildly reduced ejection fraction (HFmrEF), evidence is more limited, though SGLT2 inhibitors have shown benefit in this population as well. In heart failure with preserved ejection fraction (HFpEF), therapeutic options have historically been limited, but SGLT2 inhibitors have recently demonstrated reductions in heart failure hospitalization and cardiovascular death, representing a major therapeutic advance. Although detailed echocardiographic data were unavailable, exploratory subgroup analyses were performed using ICD-coded surrogate phenotypes for HFrEF and HFpEF. These analyses were broadly consistent with the primary findings, although notably, all-cause hospitalization was significantly higher with dual therapy in both the HFrEF (HR 1.49, 95% CI 1.13 to 1.97) and HFpEF (HR 1.79, 95% CI 1.20 to 2.68) subgroups, representing a divergence from the primary analysis that warrants cautious interpretation. These subgroup findings should be interpreted cautiously, given the limitations of administrative coding. This represents an important limitation when interpreting findings in the context of current guideline recommendations [15,16].

The observed associations of dual SGLT2 inhibitor and GLP-1 analogue therapy likely reflect complementary but distinct mechanisms of action. SGLT2 inhibitors reduce glucose reabsorption in the proximal renal tubule, leading to glycosuria, osmotic diuresis, and natriuresis, which reduces preload and afterload while improving myocardial energetics and reducing oxidative stress. Beyond these hemodynamic effects, SGLT2 inhibitors exert important pleiotropic actions, including attenuation of cardiac fibrosis, improvement of mitochondrial function, reduction in oxidative stress, and modulation of myocardial sodium-calcium handling, which may collectively contribute to reverse cardiac remodeling independent of glycemic control [17,18]. GLP-1 analogues, in contrast, enhance glucose-dependent insulin secretion, suppress glucagon release, slow gastric emptying, and promote satiety-mediated weight loss [19,20]. Beyond glycemic control, GLP-1 analogues exert direct cardiovascular effects including improved endothelial function, reduced inflammation, and favorable modulation of blood pressure and lipid profiles [21,22]. These pleiotropic properties, encompassing anti-inflammatory, anti-atherosclerotic, and vasodilatory effects, may extend the cardiovascular benefit of GLP-1 receptor agonism beyond what would be expected from glucose lowering alone, and may contribute to the clinical endpoints observed in the present study [19,20,21,22]. However, despite biologically complementary mechanisms, overlapping pleiotropic pathways may limit additive clinical benefit on hard cardiovascular outcomes, which may partly explain the absence of significant differences in major cardiovascular endpoints in the overall cohort. Additionally, differences in healthcare engagement and treatment intensity between groups may have contributed to the observed outcomes, as patients receiving dual therapy may be more closely monitored and receive more comprehensive cardiometabolic management.

These findings are consistent with evidence from major cardiovascular outcome trials evaluating each drug class individually. The EMPA-REG OUTCOME trial by Zinman et al. demonstrated a significant reduction in all-cause and cardiovascular mortality in patients receiving empagliflozin with type 2 diabetes and established cardiovascular disease [23]. Similarly, the CANVAS Program by Neal et al. and DECLARE–TIMI 58 trials by Wiviott et al. demonstrated a reduction in heart failure hospitalization and renal outcomes with canagliflozin and dapagliflozin, respectively [24,25]. Moreover, studies including GLP-1 receptor agonists, the LEADER trial (liraglutide) and SUSTAIN-6 trial by Marso et al. demonstrated significant reductions in major adverse cardiovascular events, particularly myocardial infarction and stroke among patients with type 2 diabetes [26,27]. Similarly, the REWIND trial further supports cardiovascular benefit in middle-aged adults and patients with type 2 diabetes [28]. More recent heart failure trials including DAPA-HF, EMPEROR-Reduced, EMPEROR-Preserved, and DELIVER further established the role of SGLT2 inhibitors across the spectrum of heart failure phenotypes independent of diabetes status, while STEP-HFpEF demonstrated symptomatic improvement with semaglutide in obese patients with HFpEF [29,30,31,32,33]. Although no large randomized trial has directly evaluated the combination of SGLT2 inhibitors and GLP-1 receptor agonists, the present findings should not be interpreted as evidence of superiority or inferiority of combination therapy, and indirect comparisons with randomized controlled trials should therefore be interpreted cautiously due to differences in populations, endpoints, and study design.

Our findings also align with large real-world observational studies evaluating cardiovascular and renal outcomes associated with SGLT2 inhibitors and GLP-1 receptor agonists. The CVD-REAL study demonstrated that initiation of SGLT2 inhibitors was associated with lower rates of heart failure hospitalization and all-cause mortality compared with other glucose-lowering therapies across multiple countries, regardless of pre-existing cardiovascular illness [34], although residual confounding cannot be excluded. Moreover, meta-analyses of randomized trials have shown that SGLT2 inhibitors reduce cardiovascular and renal outcomes across broad cardiovascular risk populations [35]. In contrast to prior observational analyses suggesting broad cardiovascular benefit with combination therapy, the present findings did not demonstrate significant reductions in mortality or major cardiovascular outcomes in the overall matched cohort. However, subgroup analyses demonstrated lower mortality among younger patients and women receiving dual therapy, findings that are biologically plausible given the demographic enrichment observed in prior GLP-1 receptor agonist cardiovascular outcome trials. These subgroup findings should be interpreted cautiously and considered hypothesis-generating.

Dual therapy was also associated with a modest increase in laboratory-defined hypoglycemia (glucose ≤ 70 mg/dL). Although both SGLT2 inhibitors and GLP-1 receptor agonists individually have relatively low intrinsic hypoglycemia risk, additive glucose-lowering effects in patients receiving concurrent insulin or sulfonylurea therapy may contribute to asymptomatic biochemical hypoglycemia. Importantly, the present outcome definition was laboratory-based rather than symptom-based and therefore may capture clinically mild events. Furthermore, differential laboratory surveillance intensity between groups could plausibly contribute to this association, particularly in patients receiving more intensive diabetes management and monitoring protocols [19,36]. In the conservative analysis, we did not observe a significant difference in urinary tract infection rates between dual therapy and SGLT2 inhibitor monotherapy. This contrasts with our prior expectation that the glycosuria-mediated UTI risk of SGLT2 inhibitors might dominate. Possible explanations include attenuation of glycosuria by improved overall glycemic control in patients on combined GLP-1 RA therapy, weight loss reducing the obesity-associated UTI risk component, or simply a true null effect. Despite the known association between SGLT2 inhibitors and increased glycosuria, which theoretically may predispose to urinary tract infections, existing evidence suggests that this risk does not consistently translate into higher rates of clinically significant urinary tract infections. Systematic reviews and meta-analyses of randomized controlled trials have demonstrated no significant overall increase in urinary tract infection risk with SGLT2 inhibitor therapy [37].

Unlike other outcomes that were largely neutral, retinopathy represented the strongest and most robust safety signal in the present study, remaining statistically significant after Bonferroni adjustment for multiple comparisons. Although surveillance bias and differential ophthalmologic monitoring among patients receiving GLP-1 receptor agonists may contribute, residual confounding alone is unlikely to completely account for the finding. Even though causality cannot be established from an observational study, this finding deserves attention due to its robustness relative to the other evaluated outcomes. Rapid glycemic improvement has previously been associated with transient worsening of diabetic retinopathy, particularly in semaglutide-containing regimens, and may represent one biologically plausible mechanism [27,38]. Prior analyses from the SUSTAIN-6 trial suggested that the magnitude and rapidity of HbA1c reduction may contribute to early worsening of diabetic retinopathy in susceptible individuals with pre-existing disease and poor baseline glycemic control [27,38]. Accordingly, this observation should be considered a hypothesis-generating safety signal warranting prospective evaluation rather than evidence of causality.

### Limitations

Several limitations should be acknowledged. First, the observational design precludes causal inference despite propensity score matching. Second, use of the TriNetX database introduces potential misclassification and coding inaccuracies related to diagnoses, comorbidities, medication exposure, and outcomes, as ascertainment relied on ICD-10 codes without manual validation. Third, incomplete availability of heart failure phenotyping by left ventricular ejection fraction (HFrEF, HFmrEF, HFpEF) limited subgroup-specific interpretation and mechanistic insights. Fourth, important clinical variables—including left ventricular ejection fraction, NYHA class, NT-proBNP, echocardiographic parameters, medication adherence, and longitudinal treatment changes—were inconsistently available, limiting adjustment for disease severity and heterogeneity. Fifth, exposure misclassification remains possible because prescription overlap does not confirm adherence, persistence, switching patterns, treatment duration, or dosage intensity. Sixth, residual confounding, including confounding by indication and treatment-intensity bias, remains possible despite E-value analysis and use of a negative control outcome (appendicitis), as unmeasured confounders cannot be excluded. Seventh, multiple outcome testing increases the risk of type I error; although Bonferroni and Benjamini–Hochberg corrections were applied, only retinopathy remained significant after Bonferroni adjustment. Eighth, violations of proportional hazards assumptions for acute myocardial infarction and new-onset diuretic use suggest reported hazard ratios may reflect time-varying effects and should be interpreted cautiously. Ninth, the increased incidence of retinopathy and laboratory-defined hypoglycemia in the dual therapy group may reflect residual confounding, surveillance bias, differential monitoring intensity, or rapid glycemic improvement rather than direct drug effects. Finally, rare adverse events may be underreported in electronic health record databases, and some statistically significant findings may represent modest absolute clinical effects despite relative differences between groups.

## 5. Conclusions

In a propensity score-matched real-world cohort of patients with heart failure, dual SGLT2 inhibitor and GLP-1 receptor agonist therapy was not associated with significant reductions in all-cause mortality, hospitalization, or major cardiovascular outcomes compared with SGLT2 inhibitor monotherapy over 365 days of follow-up. These findings are clinically relevant because they suggest that the addition of GLP-1 receptor agonists to established SGLT2 inhibitor therapy may not provide substantial short-term gains in major heart failure outcomes in routine clinical practice. However, subgroup analyses suggested significantly higher all-cause hospitalization with dual therapy among patients with HFrEF and HFpEF, findings that warrant cautious interpretation given the observational design. Subgroup analyses suggested lower mortality among younger patients and women, while retinopathy, laboratory-defined hypoglycemia, and pulmonary edema emerged as potential safety signals. These findings should be interpreted cautiously given the retrospective observational design and should be considered hypothesis-generating rather than causal. Prospective randomized studies are needed before clinical adoption of combination therapy specifically for heart failure outcomes.

## Figures and Tables

**Table 1:** Baseline Characteristics.

Characteristic	SGLT2 Inhibitors and GLP Analogues (*N* = 3426)	SGLT2 Inhibitors Only (*N* = 103,030)	Std Diff.	SGLT2 Inhibitors and GLP Analogues (*N* = 3421)	SGLT2 Inhibitors Only (*N* = 3421)	Std Diff.
	Before PSM			After PSM		
Demographics						
Current Age	66.0 ± 12.4	69.7 ± 13.8	0.281	66.0 ± 12.4	66.3 ± 13.5	0.026
Age at Index	62.6 ± 12.4	67.0 ± 14.0	0.335	62.6 ± 12.4	63.0 ± 13.5	0.031
White	1932 (56.40%)	56,035 (54.40%)	0.040	1928 (56.40%)	2017 (59.00%)	0.053
Unknown Race	427 (12.50%)	19,738 (19.20%)	0.184	427 (12.50%)	392 (11.50%)	0.032
Female	1251 (36.50%)	37,562 (36.50%)	0.001	1249 (36.50%)	1275 (37.30%)	0.016
Unknown Ethnicity	888 (25.90%)	29,830 (29.00%)	0.068	885 (25.90%)	938 (27.40%)	0.035
Not Hispanic or Latino	2272 (66.30%)	67,840 (65.80%)	0.010	2270 (66.40%)	2251 (65.80%)	0.012
Hispanic or Latino	266 (7.80%)	5360 (5.20%)	0.104	266 (7.80%)	232 (6.80%)	0.038
Black or African American	670 (19.60%)	16,931 (16.40%)	0.081	670 (19.60%)	632 (18.50%)	0.028
Male	2175 (63.50%)	65,468 (63.50%)	0.001	2172 (63.50%)	2146 (62.70%)	0.016
Asian	185 (5.40%)	5772 (5.60%)	0.009	184 (5.40%)	177 (5.20%)	0.009
Diagnosis						
Diabetes mellitus	2024 (59.10%)	35,991 (34.90%)	0.499	2019 (59.00%)	1840 (53.80%)	0.106
Overweight, obesity and other hyperalimentation	1094 (31.90%)	20,326 (19.70%)	0.282	1091 (31.90%)	931 (27.20%)	0.103
Malnutrition	81 (2.40%)	4557 (4.40%)	0.114	81 (2.40%)	65 (1.90%)	0.032
Hypertensive heart disease	1064 (31.10%)	33,507 (32.50%)	0.031	1063 (31.10%)	931 (27.20%)	0.085
Ischemic heart diseases	1492 (43.50%)	49,613 (48.20%)	0.093	1489 (43.50%)	1333 (39.00%)	0.093
Atherosclerosis	214 (6.20%)	6698 (6.50%)	0.010	214 (6.30%)	201 (5.90%)	0.016
Peripheral vascular disease, unspecified	155 (4.50%)	5160 (5.00%)	0.023	155 (4.50%)	110 (3.20%)	0.068
Other secondary pulmonary hypertension	348 (10.20%)	13,154 (12.80%)	0.082	348 (10.20%)	298 (8.70%)	0.050
Cerebral infarction	195 (5.70%)	5558 (5.40%)	0.013	194 (5.70%)	181 (5.30%)	0.017
Sequelae of cerebrovascular disease	85 (2.50%)	2497 (2.40%)	0.004	85 (2.50%)	75 (2.20%)	0.019
Systolic (congestive) heart failure	1184 (34.60%)	42,314 (41.10%)	0.135	1184 (34.60%)	1062 (31.00%)	0.076
Diastolic (congestive) heart failure	588 (17.20%)	16,061 (15.60%)	0.043	586 (17.10%)	507 (14.80%)	0.063
Atrial fibrillation and flutter	700 (20.40%)	29,571 (28.70%)	0.193	700 (20.50%)	644 (18.80%)	0.041
Cardiomyopathy	536 (15.60%)	23,280 (22.60%)	0.177	535 (15.60%)	476 (13.90%)	0.049
Nonrheumatic mitral valve disorders	305 (8.90%)	13,349 (13.00%)	0.130	304 (8.90%)	263 (7.70%)	0.043
Nonrheumatic aortic valve disorders	197 (5.80%)	8811 (8.60%)	0.109	197 (5.80%)	180 (5.30%)	0.022
Nonrheumatic tricuspid valve disorders	148 (4.30%)	4788 (4.60%)	0.016	146 (4.30%)	125 (3.70%)	0.031
Nonrheumatic pulmonary valve disorders	51 (1.50%)	1407 (1.40%)	0.010	49 (1.40%)	33 (1.00%)	0.043
Endocarditis, valve unspecified	28 (0.80%)	880 (0.90%)	0.004	28 (0.80%)	23 (0.70%)	0.017
Hypo-osmolality and hyponatremia	267 (7.80%)	8961 (8.70%)	0.033	267 (7.80%)	273 (8.00%)	0.007
Hyperkalemia	197 (5.80%)	5093 (4.90%)	0.036	196 (5.70%)	146 (4.30%)	0.067
Disorders of thyroid gland	355 (10.40%)	11,970 (11.60%)	0.04	354 (10.30%)	324 (9.50%)	0.029
Other chronic obstructive pulmonary disease	351 (10.20%)	13,953 (13.50%)	0.102	350 (10.20%)	295 (8.60%)	0.055
Diseases of liver	245 (7.20%)	8946 (8.70%)	0.057	245 (7.20%)	216 (6.30%)	0.034
Chronic kidney disease (CKD)	661 (19.30%)	19,801 (19.20%)	0.002	660 (19.30%)	576 (16.80%)	0.064
Procedure						
Echocardiography Procedures	1102 (32.20%)	39,646 (38.50%)	0.132	1100 (32.20%)	985 (28.80%)	0.073
Cardiac Catheterization Procedures	503 (14.70%)	14,744 (14.30%)	0.011	502 (14.70%)	437 (12.80%)	0.055
Electrocardiogram, routine ECG with at least 12 leads	1483 (43.30%)	50,871 (49.40%)	0.122	1481 (43.30%)	1306 (38.20%)	0.104
Medication						
Diuretics	1554 (45.40%)	60,385 (58.60%)	0.268	1552 (45.40%)	1323 (38.70%)	0.136
Beta blockers/related	1522 (44.40%)	56,255 (54.60%)	0.205	1518 (44.40%)	1350 (39.50%)	0.100
Angiotensin ii inhibitor	966 (28.20%)	31,734 (30.80%)	0.057	965 (28.20%)	825 (24.10%)	0.093
Ace inhibitors	565 (16.50%)	18,334 (17.80%)	0.035	562 (16.40%)	512 (15.00%)	0.040
Calcium channel blockers	885 (25.80%)	31,569 (30.60%)	0.107	884 (25.80%)	784 (22.90%)	0.068
Nitroglycerin	619 (18.10%)	22,340 (21.70%)	0.091	618 (18.10%)	585 (17.10%)	0.025
Digitalis glycosides	102 (3.00%)	4239 (4.10%)	0.062	102 (3.00%)	80 (2.30%)	0.04
Antilipemic agents	1564 (45.70%)	48,658 (47.20%)	0.032	1560 (45.60%)	1402 (41.00%)	0.093
Antiarrhythmics	1088 (31.80%)	39,734 (38.60%)	0.143	1087 (31.80%)	972 (28.40%)	0.073
Anticoagulants	1624 (47.40%)	61,459 (59.70%)	0.247	1621 (47.40%)	1424 (41.60%)	0.116
Platelet aggregation inhibitors	1337 (39.00%)	44,088 (42.80%)	0.077	1332 (38.90%)	1201 (35.10%)	0.079
Adrenal corticosteroids	688 (20.10%)	30,108 (29.20%)	0.213	687 (20.10%)	617 (18.00%)	0.052
Insulin	1573 (45.90%)	29,636 (28.80%)	0.36	1568 (45.80%)	1409 (41.20%)	0.094
Metformin	593 (17.30%)	8417 (8.20%)	0.277	591 (17.30%)	546 (16.00%)	0.035
Omeprazole	176 (5.10%)	6897 (6.70%)	0.066	176 (5.10%)	132 (3.90%)	0.062
Pantoprazole	633 (18.50%)	21,543 (20.90%)	0.061	632 (18.50%)	548 (16.00%)	0.065
Laboratory						
Sodium	2090 (61.00%)	77,888 (75.60%)	0.058	2090 (61.10%)	1877 (54.90%)	0.054
Sodium 0–0 mmol/L	137.4 ± 3.7	137.7 ± 5.1	0.327	137.4 ± 3.8	137.6 ± 4.2	0.125
Potassium	2064 (60.20%)	77,350 (75.10%)	0.079	2064 (60.30%)	1860 (54.40%)	0.080
Potassium 0–0 mmol/L	4.1 ± 0.5	4.1 ± 0.6	0.322	4.1 ± 0.5	4.1 ± 0.5	0.121
Chloride	2055 (60.00%)	72,628 (70.50%)	0.037	2055 (60.10%)	1861 (54.40%)	0.016
Chloride 0–0 mmol/L	101.3 ± 5.8	100.9 ± 12.3	0.222	101.3 ± 5.8	101.4 ± 8.7	0.115
Bicarbonate	2020 (59.00%)	70,727 (68.60%)	0.022	2020 (59.00%)	1814 (53.00%)	0.014
Bicarbonate 0–0 mmol/L	25.8 ± 4.3	25.7 ± 4.3	0.203	25.8 ± 4.4	25.7 ± 4.2	0.122
Creatinine	2038 (59.50%)	75,074 (72.90%)	0.105	2038 (59.60%)	1837 (53.70%)	0.048
Creatinine 0–0 mg/dL	1.4 ± 4.7	2.2 ± 10.7	0.286	1.4 ± 4.8	1.7 ± 7.8	0.118
Calcium	1962 (57.30%)	68,574 (66.60%)	0.085	1962 (57.40%)	1757 (51.40%)	0.050
Calcium 0–0 mg/dL	9.0 ± 0.7	8.9 ± 0.6	0.208	9.0 ± 0.8	8.9 ± 0.8	0.116
Magnesium	1413 (41.20%)	50,097 (48.60%)	0.091	1413 (41.30%)	1255 (36.70%)	0.135
Magnesium 0–0 mg/dL	2.0 ± 0.3	2.0 ± 0.3	0.150	2.0 ± 0.3	2.0 ± 0.3	0.095
Hemoglobin	2004 (58.50%)	75,923 (73.70%)	0.060	2004 (58.60%)	1799 (52.60%)	0.020
Hemoglobin 0–0 g/dL	12.4 ± 2.5	12.3 ± 2.6	0.325	12.4 ± 2.6	12.4 ± 2.7	0.121
Alanine aminotransferase	1850 (54.00%)	69,200 (67.20%)	0.044	1850 (54.10%)	1646 (48.10%)	0.008
Alanine aminotransferase 0–0 U/L	38.6 ± 76.7	43.3 ± 132.5	0.272	38.6 ± 76.7	39.2 ± 76.3	0.120
Aspartate aminotransferase	1826 (53.30%)	67,129 (65.20%)	0.051	1826 (53.40%)	1613 (47.10%)	0.022
Aspartate aminotransferase 0–0 U/L	38.3 ± 79.7	44.6 ± 155.4	0.243	38.3 ± 79.7	40.5 ± 118.6	0.125
Gamma glutamyl transferase	117 (3.40%)	6664 (6.50%)	0.062	117 (3.40%)	121 (3.50%)	0.192
Gamma glutamyl transferase 0–0 U/L	103.5 ± 134.8	94.2 ± 165.5	0.141	103.5 ± 134.8	139.6 ± 229.5	0.006
Bilirubin.total	1744 (50.90%)	63,135 (61.30%)	0.144	1744 (51.00%)	1548 (45.20%)	0.111
Bilirubin.total 0–0 mg/dL	0.7 ± 0.1	1.3 ± 5.6	0.216	0.7 ± 0.1	1.0 ± 3.5	0.112
Albumin	1815 (53.00%)	65,385 (63.50%)	0.031	1815 (53.10%)	1615 (47.20%)	0.003
Albumin 0–0 g/dL	3.6 ± 0.6	3.6 ± 0.6	0.214	3.6 ± 0.6	3.6 ± 0.6	0.117
Natriuretic peptide.B prohormone N-Terminal	630 (18.40%)	26,007 (25.20%)	0.245	630 (18.40%)	551 (16.10%)	0.068
Natriuretic peptide.B prohormone N-Terminal 0–0 pg/mL	3915.3 ± 6440.2	5646.0 ± 7658.7	0.174	3915.3 ± 6440.2	4352.6 ± 6362.3	0.060
Hemoglobin A1c/Hemoglobin.total	1535 (44.80%)	44,119 (42.80%)	0.860	1532 (44.80%)	1403 (41.00%)	0.390
Hemoglobin A1c/Hemoglobin.total 0–0%	8.5 ± 2.4	6.7 ± 1.9	0.040	8.5 ± 2.4	7.6 ± 2.2	0.076
Thyrotropin	1018 (29.70%)	40,127 (38.90%)	0.067	1018 (29.80%)	915 (26.70%)	0.039
Thyrotropin 0–0 m[IU]/L	3.0 ± 6.4	4.4 ± 29.5	0.197	3.0 ± 6.4	3.9 ± 32.7	0.068
Urea nitrogen	1991 (58.10%)	71,027 (68.90%)	0.144	1991 (58.20%)	1795 (52.50%)	0.180
Urea nitrogen 0–0 mg/dL	24.7 ± 14.6	22.8 ± 12.1	0.226	24.7 ± 14.6	22.3 ± 12.9	0.115
Cholesterol in LDL	1171 (34.20%)	41,429 (40.20%)	0.066	1168 (34.10%)	1077 (31.50%)	0.049
Cholesterol in LDL 0–0 mg/dL	89.7 ± 43.9	86.9 ± 38.8	0.125	89.6 ± 43.9	87.5 ± 41.6	0.057
Troponin I.cardiac	288 (8.40%)	10,073 (9.80%)	0.041	288 (8.40%)	251 (7.30%)	0.081
Troponin I.cardiac 0–0 ng/mL	3.6 ± 13.9	4.5 ± 24.9	0.048	3.6 ± 13.9	5.4 ± 28.1	0.040
BMI	1725 (50.40%)	61,448 (59.60%)	0.579	1725 (50.40%)	1541 (45.00%)	0.235
BMI 0–0 kg/m^2^	35.0 ± 9.3	30.1 ± 8.1	0.184	35.0 ± 9.3	33.0 ± 8.6	0.111
Cholesterol	1163 (33.90%)	41,845 (40.60%)	0.124	1163 (34.00%)	1076 (31.50%)	0.069
Cholesterol 0–0 mg/dL	157.2 ± 53.9	150.7 ± 51.4	0.138	157.2 ± 53.9	153.5 ± 52.1	0.054
Left Ventricular Ejection Fraction (LVEF)	230 (6.70%)	12,042 (11.70%)	0.272	230 (6.70%)	219 (6.40%)	0.097
LVEF 0–0%	43.3 ± 16.4	38.9 ± 16.2	0.173	43.3 ± 16.4	41.7 ± 16.8	0.013
Body weight	1681 (49.10%)	60,423 (58.60%)	0.496	1680 (49.10%)	1500 (43.80%)	0.212
Body weight 0–0 lb	223.8 ± 67.5	192.8 ± 57.4	0.193	223.8 ± 67.5	210.4 ± 59.2	0.106

**Table 2:** Outcomes at 1 Year Follow-up.

Outcome	SGLT2 Inhibitors and GLP Analogues (*n*/*N*)	SGLT2 Inhibitors Only (*n*/*N*)	HR (95% CI)	Log-Rank *p* Value
All-Cause Mortality				
At 1 Year	250/3401	267/3407	0.92 (0.77–1.09)	0.343
AMI				
At 1 Year	124/2589	105/2653	1.20 (0.92–1.55)	0.176
AKF				
At 1 Year	252/2405	247/2555	1.05 (0.89–1.26)	0.554
Incident AF				
At 1 Year	172/2394	163/2450	1.05 (0.85–1.30)	0.650
All-Cause Hospitalization				
At 1 Year	230/892	192/866	1.16 (0.96–1.40)	0.134
Pulmonary Edema				
At 1 Year	163/3079	121/3119	1.35 (1.06–1.70)	0.013
UTI				
At 1 Year	137/3150	115/3126	1.16 (0.90–1.48)	0.253
New Onset Diuretic				
At 1 Year	237/631	297/755	0.92 (0.78–1.09)	0.354
Hypoglycemia				
At 1 Year	323/3145	261/3132	1.22 (1.04–1.44)	0.015
Retinopathy				
At 1 Year	93/3232	35/3298	2.66 (1.81–3.93)	0.000

HR = Hazard Ratio; CI = Confidence Interval, *n* = population with outcome, *N* = total population of cohort. Denominators vary across outcomes due to outcome-specific cohort definitions, eligibility criteria, and variable data availability within the TriNetX database.

## Data Availability

The data that support the findings of this study are available from the TriNetX Research Network. Due to licensing restrictions and data use agreements, the raw data are not publicly available. Access to the TriNetX platform can be obtained through institutional subscription. Aggregate data supporting the findings of this study may be available from the corresponding author upon reasonable request and with permission from TriNetX.

## Supplementary Materials

The following supporting information can be downloaded at: https://www.mdpi.com/article/10.3390/biomedicines14061368/s1, Table S1. ICD Codes and Outcome Definitions; Table S2. Criteria for Cohort SGLT2 inhibitors and GLP-1 analogs; Table S3. Criteria for Cohort SGLT2 inhibitors only; Table S4. HFrEF Subgroup Analysis; Table S5. HFpEF Subgroup Analysis; Table S6. Age Subgroup Analyses (18–64 and ≥65); Table S7. Gender Subgroup Analyses; Table S8. Bonferroni p and BH-FDR q values for all outcomes at 1-Year; Table S9. E-values for Statistically Significant Outcomes; Table S10. Proportional Hazard (PH) Assumption Test for all Outcomes; Table S11. Absolute Risk Increase (ANI) and Number Needed to Harm (NNH) for Significant Outcomes; Figure S1. Propensity Score Matching; Figure S2. Kaplan–Meier curves for All-cause Hospitalization at 1-Year; Figure S3: Kaplan–Meier curves for Acute Myocardial Infarction at 1-Year; Figure S4. Kaplan–Meier curves for Incident Atrial Fibrillation at 1-Year; Figure S5. Kaplan–Meier curves for Acute Kidney Failure at 1-Year; Figure S6. Kaplan–Meier curves for Pulmonary Edema at 1-Year; Figure S7. Kaplan–Meier curves for New Onset Diuretics at 1-Year; Figure S8. Kaplan–Meier curves for Urinary Tract Infection at 1-Year; Figure S9. Kaplan–Meier curves for Retinopathy at 1-Year; Figure S10. Kaplan–Meier curves for Hypoglycemia at 1-Year.

## References

[1] Savarese G., Becher P.M., Lund L.H., Seferovic P., Rosano G.M.C., Coats A.J.S. (2022). Global burden of heart failure: A comprehensive and updated review of epidemiology. Cardiovasc. Res..

[2] Heidenreich P.A., Fonarow G.C., Opsha Y., Sandhu A.T., Sweitzer N.K., Warraich H.J. (2022). Economic issues in heart failure in the United States. J. Card. Fail..

[3] Chen W., Heidenreich P.A., Sandhu A.T. (2024). The economics of heart failure care. Prog. Cardiovasc. Dis..

[4] Ziaeian B., Fonarow G.C. (2016). Epidemiology and aetiology of heart failure. Nat. Rev. Cardiol..

[5] Chen K., Nie Z., Shi R., Yu D., Wang Q., Shao F., Wu G., Wu Z., Chen T., Li C. (2023). Time to benefit of sodium–glucose cotransporter-2 inhibitors among patients with heart failure. JAMA Netw. Open.

[6] Anker S.D., Usman M.S., Butler J. (2022). SGLT2 Inhibitors: From Antihyperglycemic Agents to All-Around Heart Failure Therapy. Circulation.

[7] Abdelhamid M., Abdrabou M.M., Faris E., El Kafas S., Hosny M., Hassan A. (2025). Glucagon-like Peptide-1 Receptor Agonists in Heart Failure: Mechanisms, Evidence and Identifying Optimal Candidates. Card. Fail. Rev..

[8] Yang H.M. (2025). GLP-1 agonists in cardiovascular diseases: Mechanisms, clinical evidence, and emerging therapies. J. Clin. Med..

[9] Khalid A., Rodriguez P., Tasouli-Drakou V., Ahmed A.-B., Thatcher S., Dugal J.K., Singh A. (2025). Breaking new ground in heart failure management: Novel therapies and future frontiers. Front. Cardiovasc. Med..

[10] Colombijn J.M.T., de Leijer J.F., Visseren F.L.J., Verhaar M.C., van Raalte D.H., Sattar N., Vernooij R.W.M., van Sloten T.T. (2026). Effectiveness and safety of combining SGLT2 inhibitors and GLP-1 receptor agonists in individuals with type 2 diabetes: A systematic review and meta-analysis of cohort studies. Diabetologia.

[11] Shokravi A., Seth J., Mancini G.B.J. (2025). Cardiovascular and renal outcomes of dual combination therapies with glucagon-like peptide-1 receptor agonists and sodium-glucose transport protein 2 inhibitors: A systematic review and meta-analysis. Cardiovasc. Diabetol..

[12] Austin P.C. (2011). Optimal caliper widths for propensity-score matching when estimating differences in means and differences in proportions in observational studies. Pharm. Stat..

[13] D’Agostino R.B. (2007). Propensity Scores in Cardiovascular Research. Circulation.

[14] Sashegyi A., Ferry D. (2017). On the interpretation of the hazard ratio and communication of survival benefit. Oncologist.

[15] McDonagh T.A., Metra M., Adamo M., Gardner R.S., Baumbach A., Böhm M., Burri H., Butler J., Čelutkienė J., Chioncel O. (2023). 2023 Focused Update of the 2021 ESC Guidelines for the Diagnosis and Treatment of Acute and Chronic Heart Failure: Developed by the Task Force for the Diagnosis and Treatment of Acute and Chronic Heart Failure of the European Society of Cardiology (ESC) with the Special Contribution of the Heart Failure Association (HFA) of the ESC. Eur. Heart J..

[16] Heidenreich P.A., Bozkurt B., Aguilar D., Allen L.A., Byun J.J., Colvin M.M., Deswal A., Drazner M.H., Dunlay S.M., Evers L.R. (2022). 2022 AHA/ACC/HFSA Guideline for the Management of Heart Failure. J. Am. Coll. Cardiol..

[17] Heerspink H.J.L., Perkins B.A., Fitchett D.H., Husain M., Cherney D.Z.I. (2016). Sodium Glucose Cotransporter 2 Inhibitors in the Treatment of Diabetes Mellitus. Circulation.

[18] Verma S., McMurray J.J.V. (2018). SGLT2 inhibitors and mechanisms of cardiovascular benefit: A state-of-the-art review. Diabetologia.

[19] Drucker D.J., Nauck M.A. (2006). The incretin system: Glucagon-like peptide-1 receptor agonists and dipeptidyl peptidase-4 inhibitors in type 2 diabetes. Lancet.

[20] Müller T.D., Finan B., Bloom S.R., D’Alessio D., Drucker D.J., Flatt P.R., Fritsche A., Gribble F., Grill H.J., Habener J.F. (2019). Glucagon-like peptide 1 (GLP-1). Mol. Metab..

[21] Lovshin J.A., Drucker D.J. (2009). Incretin-based therapies for type 2 diabetes mellitus. Nat. Rev. Endocrinol..

[22] Nauck M.A., Meier J.J. (2018). Incretin hormones: Their role in health and disease. Diabetes Obes. Metab..

[23] Zinman B., Wanner C., Lachin J.M., Fitchett D., Bluhmki E., Hantel S., Mattheus M., Devins T., Johansen O.E., Woerle H.J. (2015). Empagliflozin, cardiovascular outcomes, and mortality in type 2 diabetes. N. Engl. J. Med..

[24] Neal B., Perkovic V., Mahaffey K.W., De Zeeuw D., Fulcher G., Erondu N., Shaw W., Law G., Desai M., Matthews D.R. (2017). Canagliflozin and cardiovascular and renal events in type 2 diabetes. N. Engl. J. Med..

[25] Wiviott S.D., Raz I., Bonaca M.P., Mosenzon O., Kato E.T., Cahn A., Silverman M.G., Zelniker T.A., Kuder J.F., Murphy S.A. (2019). Dapagliflozin and cardiovascular outcomes in type 2 diabetes. N. Engl. J. Med..

[26] Marso S.P., Daniels G.H., Brown-Frandsen K., Kristensen P., Mann J.F.E., Nauck M.A., Nissen S.E., Pocock S., Poulter N.R., Ravn L.S. (2016). Liraglutide and cardiovascular outcomes in type 2 diabetes. N. Engl. J. Med..

[27] Marso S.P., Bain S.C., Consoli A., Eliaschewitz F.G., Jódar E., Leiter L.A., Lingvay I., Rosenstock J., Seufert J., Warren M.L. (2016). Semaglutide and cardiovascular outcomes in patients with type 2 diabetes. N. Engl. J. Med..

[28] Gerstein H.C., Colhoun H.M., Dagenais G.R., Diaz R., Lakshmanan M., Pais P., Probstfield J., Riesmeyer J.S., Riddle M.C., Rydén L. (2019). Dulaglutide and cardiovascular outcomes in type 2 diabetes (REWIND). Lancet.

[29] McMurray J.J.V., Solomon S.D., Inzucchi S.E., Køber L., Kosiborod M.N., Martinez F.A., Ponikowski P., Sabatine M.S., Anand I.S., Bělohlávek J. (2019). Dapagliflozin in patients with heart failure and reduced ejection fraction. N. Engl. J. Med..

[30] Packer M., Anker S.D., Butler J., Filippatos G., Pocock S.J., Carson P., Januzzi J., Verma S., Tsutsui H., Brueckmann M. (2020). Cardiovascular and renal outcomes with empagliflozin in heart failure. N. Engl. J. Med..

[31] Anker S.D., Butler J., Filippatos G., Ferreira J.P., Bocchi E., Böhm M., Rocca H.-P.B., Choi D.-J., Chopra V., Chuquiure-Valenzuela E. (2021). Empagliflozin in heart failure with a preserved ejection fraction. N. Engl. J. Med..

[32] Solomon S.D., McMurray J.J.V., Claggett B., De Boer R.A., DeMets D., Hernandez A.F., Inzucchi S.E., Kosiborod M.N., Lam C.S.P., Martinez F. (2022). Dapagliflozin in heart failure with mildly reduced or preserved ejection fraction. N. Engl. J. Med..

[33] Kosiborod M.N., Abildstrøm S.Z., Borlaug B.A., Butler J., Rasmussen S., Davies M., Hovingh G.K., Kitzman D.W., Lindegaard M.L., Møller D.V. (2023). Semaglutide in Patients with Heart Failure with Preserved Ejection Fraction and Obesity. N. Engl. J. Med..

[34] Kosiborod M., Cavender M.A., Fu A.Z., Wilding J.P., Khunti K., Holl R.W., Norhammar A., Birkeland K.I., Jørgensen M.E., Thuresson M. (2017). Lower risk of heart failure and death in patients initiated on SGLT2 inhibitors versus other glucose-lowering drugs. Circulation.

[35] Zelniker T.A., Wiviott S.D., Raz I., Im K., Goodrich E.L., Bonaca M.P., Mosenzon O., Kato E.T., Cahn A., Furtado R.H.M. (2018). SGLT2 inhibitors for primary and secondary prevention of cardiovascular and renal outcomes in type 2 diabetes: A systematic review and meta-analysis of cardiovascular outcome trials. Lancet.

[36] Ja’arah D., Zoubi M.S.A., Abdelhady G., Rabi F., Tambuwala M.M. (2021). Role of glucagon-like peptide-1 receptor agonists in hypoglycemia. Clin. Med. Insights Endocrinol. Diabetes.

[37] Donnan J.R., Grandy C.A., Chibrikov E., Marra C.A., Aubrey-Bassler K., Johnston K., Swab M., Hache J., Curnew D., Nguyen H. (2019). Comparative safety of the sodium glucose co-transporter 2 (SGLT2) inhibitors: A systematic review and meta-analysis. BMJ Open.

[38] Vilsbøll T., Bain S.C., Leiter L.A., Lingvay I., Matthews D., Simó R., Helmark I.C., Wijayasinghe N., Larsen M. (2019). Semaglutide, reduction in glycated haemoglobin and the risk of diabetic retinopathy. Diabetes Obes. Metab..

[39] De Sanctis V., Soliman A.T., Daar S., Tzoulis P., Fiscina B., Kattamis C. (2022). International Network of Clinicians for Endocrinopathies in Thalassemia and Adolescence Medicine Icet-A. Retrospective observational studies: Lights and shadows for medical writers. Acta Biomed..

