# Supplementary material for: Clinical Outcomes of GLP-1 Receptor Agonist and SGLT2 Inhibitor Combination Therapy in Heart Failure: A Real-World Propensity-Matched TriNetX Analysis
Source: Biomedicines. 2026 Jun 17;14(6):1368. doi: 10.3390/biomedicines14061368 (PMC13296858; doi:10.3390/biomedicines14061368)

Supplementary Table S1. ICD Codes and Outcome Definitions

**POPULATION**		
**Demographics**	**UMLS:HL7V3.0:Gender:M**	**Male**
**Demographics**	**UMLS:HL7V3.0:Gender:F**	**Female**
**Diagnosis**	**UMLS:ICD10CM:I50**	**Heart Failure**
**DIAGNOSIS**		
**Diagnosis**	**E08-E13**	**Diabetes mellitus**
**Diagnosis**	**E65-E68**	**Overweight, obesity and other hyperalimentation**
**Diagnosis**	**E40-E46**	**Malnutrition**
**Diagnosis**	**I11**	**Hypertensive heart disease**
**Diagnosis**	**I20-I25**	**Ischemic heart diseases**
**Diagnosis**	**I70**	**Atherosclerosis**
**Diagnosis**	**I73.9**	**Peripheral vascular disease, unspecified**
**Diagnosis**	**I27.2**	**Other secondary pulmonary hypertension**
**Diagnosis**	**I63**	**Cerebral infarction**
**Diagnosis**	**I69**	**Sequelae of cerebrovascular disease**
**Diagnosis**	**I50.2**	**Systolic (congestive) heart failure**
**Diagnosis**	**I50.3**	**Diastolic (congestive) heart failure**
**Diagnosis**	**I48**	**Atrial fibrillation and flutter**
**Diagnosis**	**I42**	**Cardiomyopathy**
**Diagnosis**	**I34**	**Nonrheumatic mitral valve disorders**
**Diagnosis**	**I35**	**Nonrheumatic aortic valve disorders**
**Diagnosis**	**I36**	**Nonrheumatic tricuspid valve disorders**
**Diagnosis**	**I37**	**Nonrheumatic pulmonary valve disorders**
**Diagnosis**	**I38**	**Endocarditis, valve unspecified**
**Diagnosis**	**E87.1**	**Hypo-osmolality and hyponatremia**
**Diagnosis**	**E87.5**	**Hyperkalemia**

	Diagnosis	E00-E07	Disorders of thyroid gland
	Diagnosis	J44	Other chronic obstructive pulmonary disease
	Diagnosis	K70-K77	Diseases of liver
	Diagnosis	N18	Chronic kidney disease (CKD)
		MEDICATION	
SGLT2i			
	Medication	NLM:ATC:A10BK	Sodium glucose co-transporter 2 inhibitors
Member list			
	Medication	A10BK01	dapagliflozin
	Medication	A10BK02	canagliflozin
	Medication	A10BK03	empagliflozin
	Medication	A10BK04	ertugliflozin
	Medication	A10BK06	sotagliflozin
GLP-1 Analogs			
	Medication	NLM:ATC:A10BJ	Glucagon-like peptide-1 analogues
Member list			
	Medication	A10BJ01	Exenatide
	Medication	A10BJ02	Liraglutide
	Medication	A10BJ03	Lixisenatide
	Medication	A10BJ05	Dulaglutide
	Medication	A10BJ06	Semaglutide
		OUTCOMES	
All-Cause Mortality			
Outcome definition			
	Demographics	Deceased	Deceased
Settings for the performed analyses			
	Risk analysis		excluding patients with outcome prior to the time window
	Kaplan - Meier survival analysis		excluding patients with outcome prior to the time window
All-Cause Hospitalization			

	**Outcome definition**		
	Visit	UMLS:HL7V3.0:VisitType:EMER	Visit: Emergency
	Visit	UMLS:HL7V3.0:VisitType:ACUTE	Visit: Inpatient Acute
	Visit	UMLS:HL7V3.0:VisitType:IMP	Visit: Inpatient Encounter
	Visit	UMLS:HL7V3.0:VisitType:NONAC	Visit: Inpatient Non-acute
	Visit	UMLS:HL7V3.0:VisitType:OBSENC	Visit: Observation Encounter
	Visit	UMLS:HL7V3.0:VisitType:SS	Visit: Short Stay
	**Settings for the performed analyses**		
	Risk analysis		including patients with outcome prior to the time window
	Kaplan - Meier survival analysis		excluding patients with outcome prior to the time window
	Number of instances analysis		including patients with outcome prior to the time window<br>excluding patients with zero outcomes<br>counts are grouped by date
**incident AF**			
	**Outcome definition**		
	Diagnosis	UMLS:ICD10CM:I48	Atrial fibrillation and flutter
	**Settings for the performed analyses**		
	Number of instances analysis		excluding patients with outcome prior to the time window<br>including patients with zero outcomes<br>counts are grouped by date
	Risk analysis		excluding patients with outcome prior to the time window
	Kaplan - Meier survival analysis		excluding patients with outcome prior to the time window
**AKI**			

Outcome definition			
	Diagnosis	UMLS:ICD10CM:N17	Acute kidney failure
Settings for the performed analyses			
	Risk analysis		excluding patients with outcome prior to the time window
	Kaplan - Meier survival analysis		excluding patients with outcome prior to the time window
	Number of instances analysis		excluding patients with outcome prior to the time window<br>excluding patients with zero outcomes<br>counts are grouped by date
**New onset Diuretics**			
Outcome definition			
	Medication	NLM:ATC:C03	DIURETICS
Settings for the performed analyses			
	Number of instances analysis		excluding patients with outcome prior to the time window<br>excluding patients with zero outcomes<br>counts are grouped by date
	Kaplan - Meier survival analysis		excluding patients with outcome prior to the time window
	Risk analysis		excluding patients with outcome prior to the time window
**Hypoglycemia**			
Outcome definition			
	Laboratory	TNX:9025	Glucose [Mass/volume] in Serum, Plasma or Blood (at most 70.00 mg/dL (most recent occurrence))
Settings for the performed analyses			
	Risk analysis		excluding patients with outcome prior to the time window
	Kaplan - Meier survival analysis		excluding patients with outcome prior to the time window
	Number of instances analysis		excluding patients with outcome prior to the time window<br>excluding patients with zero outcomes<br>counts are grouped by date
**AMI**			

	**Outcome definition**			
		**Diagnosis**	**UMLS:ICD10CM:I21**	**Acute myocardial infarction**
	**Settings for the performed analyses**			
		**Risk analysis**		**excluding patients with outcome prior to the time window**
		**Number of instances analysis**		**excluding patients with outcome prior to the time window**<br>**excluding patients with zero outcomes**<br>**counts are grouped by date**
		**Kaplan - Meier survival analysis**		**excluding patients with outcome prior to the time window**
**Pulmonary edema**				
	**Outcome definition**			
		**Diagnosis**	**UMLS:ICD10CM:J81**	**Pulmonary edema**
	**Settings for the performed analyses**			
		**Kaplan - Meier survival analysis**		**excluding patients with outcome prior to the time window**
		**Risk analysis**		**excluding patients with outcome prior to the time window**
		**Number of instances analysis**		**excluding patients with outcome prior to the time window**<br>**excluding patients with zero outcomes**<br>**counts are grouped by date**
**UTI**				
	**Outcome definition**			
		**Diagnosis**	**UMLS:ICD10CM:N39.0**	**Urinary tract infection, site not specified**
	**Settings for the performed analyses**			
		**Number of instances analysis**		**excluding patients with outcome prior to the time window**<br>**excluding patients with zero outcomes**<br>**counts are grouped by date**
		**Risk analysis**		**excluding patients with outcome prior to the time window**
		**Kaplan - Meier survival analysis**		**excluding patients with outcome prior to the time window**

Retinopathy			
Outcome definition			
	Diagnosis	UMLS:ICD10CM:E11.3	Type 2 diabetes mellitus with ophthalmic complications
Settings for the performed analyses			
	Risk analysis		excluding patients with outcome prior to the time window
	Kaplan - Meier survival analysis		excluding patients with outcome prior to the time window
	Number of instances analysis		excluding patients with outcome prior to the time window<br>excluding patients with zero outcomes<br>counts are grouped by date

Supplementary Table S2. Criteria for Cohort SGLT2 inhibitors and GLP-1 analogs

Ungrouped terms				
must have		demographics	Age	Age (at least 18 years (most recent occurrence))
	and any of	demographics	UMLS:HL7V3.0:Gender:M	Male
		demographics	UMLS:HL7V3.0:Gender:F	Female
Group 1				
Group 1A HF				
must have		diagnosis	UMLS:ICD10CM:I50	Heart failure
date constraint		This group occurred before 1 year ago		

event relationship		The first instance of meds occurred within 1 month on or after the first instance of HF		
Group 1B meds				
must have		medication	NLM:ATC:A10BK	Sodium-glucose co-transporter 2 (SGLT2) inhibitors
	and	medication	NLM:ATC:A10BJ	Glucagon-like peptide-1 (GLP-1) analogues

Supplementary Table S3. Criteria for Cohort SGLT2 inhibitors only

Ungrouped terms					
	must have		demographics	Age	Age (at least 18 years (most recent occurrence))
		and any of	demographics	UMLS:HL7V3.0:Gender:M	Male
			demographics	UMLS:HL7V3.0:Gender:F	Female
	cannot have		medication	NLM:ATC:A10BJ	Glucagon-like peptide-1 (GLP-1) analogues
Group 1					
	Group 1A HF				
	must have		diagnosis	UMLS:ICD10CM:I50	Heart failure
	date constraint		This group occurred before 1 year ago		

**event relationship**	**The first instance of meds occurred within 1 month on or after the first instance of HF**		
**Group 1B meds**			
**must have**	**medication**	**NLM:ATC:A10BK**	**Sodium-glucose co-transporter 2 (SGLT2) inhibitors**

Supplementary Table S4. HFrEF Subgroup Analysis.

Outcome	SGLT2 inhibitor + GLP-1 analogue (n/N)	SGLT2 inhibitor (n/N)	HR (95% CI)	Log-rank p value
All-Cause Mortality	166/2,002	167/2,005	0.98 (0.79–1.22)	0.853
All-Cause Hospitalization	129/417	81/367	1.49 (1.13–1.97)	0.004
Acute Myocardial Infarction	69/1,364	67/1,342	1.01 (0.72–1.42)	0.941
Atrial Fibrillation and Flutter	94/1,333	91/1,356	1.03 (0.77–1.38)	0.834
Acute Kidney Injury	143/1,330	138/1,368	1.04 (0.82–1.31)	0.767
Pulmonary Edema	113/1,756	91/1,785	1.25 (0.95–1.65)	0.108
New onset diuretic use	137/318	138/346	1.09 (0.86–1.38)	0.461
Urinary Tract Infection	76/1,845	53/1,780	1.38 (0.97–1.95)	0.074

Retinopathy	47/1,887	25/1,907	1.87 (1.15–3.03)	0.01
Hypoglycemia	208/1,804	201/1,776	1.01 (0.83–1.22)	0.953

- HR = Hazard Ratio; CI = Confidence Interval
- n = population with outcome
- N = total population of cohort

Supplementary Table S5. HFpEF Subgroup Analysis.

Outcome	SGLT2 inhibitor + GLP-1 analogue (n/N)	SGLT2 inhibitor (n/N)	HR (95% CI)	Log-rank p value
All-Cause Mortality	121/1,373	103/1,373	1.17 (0.90–1.52)	0.248
All-Cause Hospitalization	719/1,380	706/1,380	1.79 (1.20–2.68)	0.004
Acute Myocardial Infarction	44/1,045	42/1,071	1.06 (0.69–1.62)	0.788
Atrial Fibrillation and Flutter	56/906	48/901	1.13 (0.77–1.66)	0.546
Acute Kidney Injury	107/832	101/905	1.14 (0.87–1.49)	0.354
Pulmonary Edema	63/1,178	43/1,212	1.52 (1.03–2.24)	0.032
New onset diuretic use	66/184	76/209	0.98 (0.70–1.36)	0.897
Urinary Tract Infection	50/1,204	48/1,196	1.03 (0.69–1.53)	0.895
Retinopathy	37/1,269	19/1,279	1.96 (1.13–3.40)	0.015
Hypoglycemia	127/1,177	99/1,166	1.28 (0.98–1.66)	0.069

- HR = Hazard Ratio; CI = Confidence Interval
- n = population with outcome
- N = total population of cohort

Supplementary Table S6. Age Subgroup Analyses (18-64 and ≥65).

Outcome	SGLT2 inhibitor + GLP-1 analogue (n/N)	SGLT2 inhibitor (n/N)	HR (95% CI)	Log-rank p value
**All-Cause Mortality**				
Age 18-64	44/1,246	67/1,246	0.64 (0.43–0.93)	0.018
Age ≥65	190/1,996	186/2,002	1.00 (0.81–1.22)	0.971
**All-Cause Hospitalization**				
Age 18-64	614/1,249	609/1,249	1.36 (0.95–1.93)	0.089
Age ≥65	906/2,013	922/2,013	1.22 (0.95–1.58)	0.121
**Acute Myocardial Infarction**				
Age 18-64	46/954	47/952	0.94 (0.63–1.42)	0.779
Age ≥65	73/1,524	66/1,546	1.12 (0.80–1.55)	0.521
**Atrial Fibrillation and Flutter**				
Age 18-64	66/1,017	45/1,029	1.41 (0.97–2.06)	0.072
Age ≥65	103/1,246	102/1,318	1.04 (0.79–1.37)	0.774

**Acute Kidney Injury**				
Age 18-64	83/917	77/940	1.05 (0.77–1.43)	0.77
Age ≥65	158/1,365	142/1,472	1.16 (0.92, 1.45)	0.21
**Pulmonary Edema**				
Age 18-64	51/1,112	40/1,127	1.26 (0.83–1.90)	0.279
Age ≥65	92/1,823	81/1,840	1.13 (0.84–1.52)	0.437
**New onset diuretic use**				
Age 18-64	80/238	99/260	0.84 (0.63–1.13)	0.262
Age ≥65	138/364	154/391	0.96 (0.76–1.20)	0.704
**Urinary Tract Infection**				
Age 18-64	37/1,159	27/1,154	1.31 (0.80–2.15)	0.287
Age ≥65	99/1,842	91/1,774	1.02 (0.76–1.35)	0.92
**Retinopathy**				
Age 18-64	37/1,181	16/1,204	2.29 (1.27–4.11)	0.004
Age ≥65	55/1,902	22/1,931	2.46 (1.50–4.03)	<0.001
**Hypoglycemia**				
Age 18-64	103/1,172	96/1,150	1.01 (0.77–1.34)	0.925

Age ≥65	203/1,829	168/1,832	1.19 (0.97–1.47)	0.088

- HR = Hazard Ratio; CI = Confidence Interval
- n = population with outcome
- N = total population of cohort

Supplementary Table S7. Gender Subgroup Analyses.

Outcome	SGLT2 inhibitor + GLP-1 analogue (n/N)	SGLT2 inhibitor (n/N)	HR (95% CI)	Log-rank p value
**All-Cause Mortality**				
Male	190/2,272	202/2,278	0.92 (0.76–1.13)	0.433
Female	71/1,283	113/1,287	0.60 (0.45–0.81)	0.001
**All-Cause Hospitalization**				
Male	1,048/2,284	1,085/2,284	1.09 (0.86–1.39)	0.483
Female	604/1,292	602/1,292	1.57 (1.13–2.18)	0.006
**Acute Myocardial Infarction**				
Male	80/1,698	74/1,748	1.11 (0.81–1.52)	0.532
Female	50/1,014	33/1,019	1.49 (0.96–2.32)	0.073
**Atrial Fibrillation and Flutter**				
Male	131/1,533	115/1,564	1.12 (0.87–1.44)	0.37

Female	51/973	60/1,000	0.83 (0.57–1.20)	0.321
**Acute Kidney Injury**				
Male	175/1,579	167/1,649	1.07 (0.86–1.32)	0.551
Female	86/939	95/965	0.87 (0.65–1.17)	0.357
**Pulmonary Edema**				
Male	121/2,068	103/2,093	1.17 (0.90–1.52)	0.236
Female	53/1,151	51/1,162	1.01 (0.69–1.49)	0.945
**New onset diuretic use**				
Male	153/443	190/489	0.84 (0.68–1.04)	0.107
Female	97/225	102/255	1.01 (0.76–1.33)	0.964
**Urinary Tract Infection**				
Male	66/2,178	65/2,160	0.98 (0.70–1.39)	0.923
Female	74/1,112	68/1,105	1.04 (0.75–1.45)	0.797
**Retinopathy**				
Male	61/2,158	28/2,214	2.20 (1.41–3.44)	<0.001
Female	39/1,224	14/1,233	2.70 (1.46–4.96)	0.001
**Hypoglycemia**				

Male	210/2,123	199/2,100	1.03 (0.85–1.25)	0.789
Female	136/1,172	113/1,164	1.16 (0.91–1.49)	0.236

- HR = Hazard Ratio; CI = Confidence Interval
- n = population with outcome
- N = total population of cohort

Supplementary Table S8. Bonferroni p and BH-FDR q values for all outcomes at 1-Year

Outcome	Bonferroni p	BH-FDR q
**All-Cause Mortaity**		
At 1-Year	1	0.443
**AMI**		
At 1-Year	1	0.352
**AKF**		
At 1-Year	1	0.616
**Incident AF**		
At 1-Year	1	0.65
**All-Cause Hospitalization**		
At 1-Year	1	0.335
**Pulmonary Edema**		
At 1-Year	0.14	0.05
**UTI**		
At 1-Year	1	0.417
**New Onset Diuretic**		
At 1-Year	1	0.443
**Hypoglycemia**		

At 1-Year	0.15	0.05
**Retinopathy**		
At 1-Year	0.001	0.001

Supplementary Table S9. E-values for Statistically Significant Outcomes

Outcome	HR (95% CI)	E-value (point)	E-value (CI)
**Hypoglycemia**			
At 1-Year	1.22 (1.04–1.44)	1.75	1.24
**Pulmonary Edema**			
At 1-Year	1.35 (1.06–1.70)	2.03	1.32
**Retinopathy**			
At 1-Year	2.66 (1.81–3.93)	4.77	3.01

Supplementary Table S10. Proportional Hazard (PH) Assumption Test for all Outcomes

Outcome	PH test p (TriNetx)
**All-Cause Mortality**	0.629 — PH holds
**All-Cause Hospitalization**	0.727 — PH holds
**Atrial Fibrillation/Flutter**	0.383 — PH holds
**Acute Kidney Failure**	0.643 — PH holds

**New-onset Diuretics**	0.030 — PH violated
**Hypoglycemia**	0.692 — PH holds
**Acute Myocardial Infarction**	0.008 — PH violated
**Pulmonary Edema**	0.456 — PH holds
**Urinary Tract Infection**	0.494 — PH holds
**Retinopathy**	0.301 — PH holds

Supplementary Table S11. Absolute Risk Increase (ANI) and Number Needed to Harm (NNH) for Significant Outcomes

Outcome	Risk Difference	ARI	NNH
**Hypoglycemia**			
At 1-Year	0.103 − 0.083 = 0.020	2.00%	50
**Pulmonary Edema**			
At 1-Year	0.053 − 0.039 = 0.014	1.40%	71
**Retinopathy**			
At 1-Year	0.029 − 0.011 = 0.018	1.80%	56

Supplementary Figure S1. Propensity Score Matching

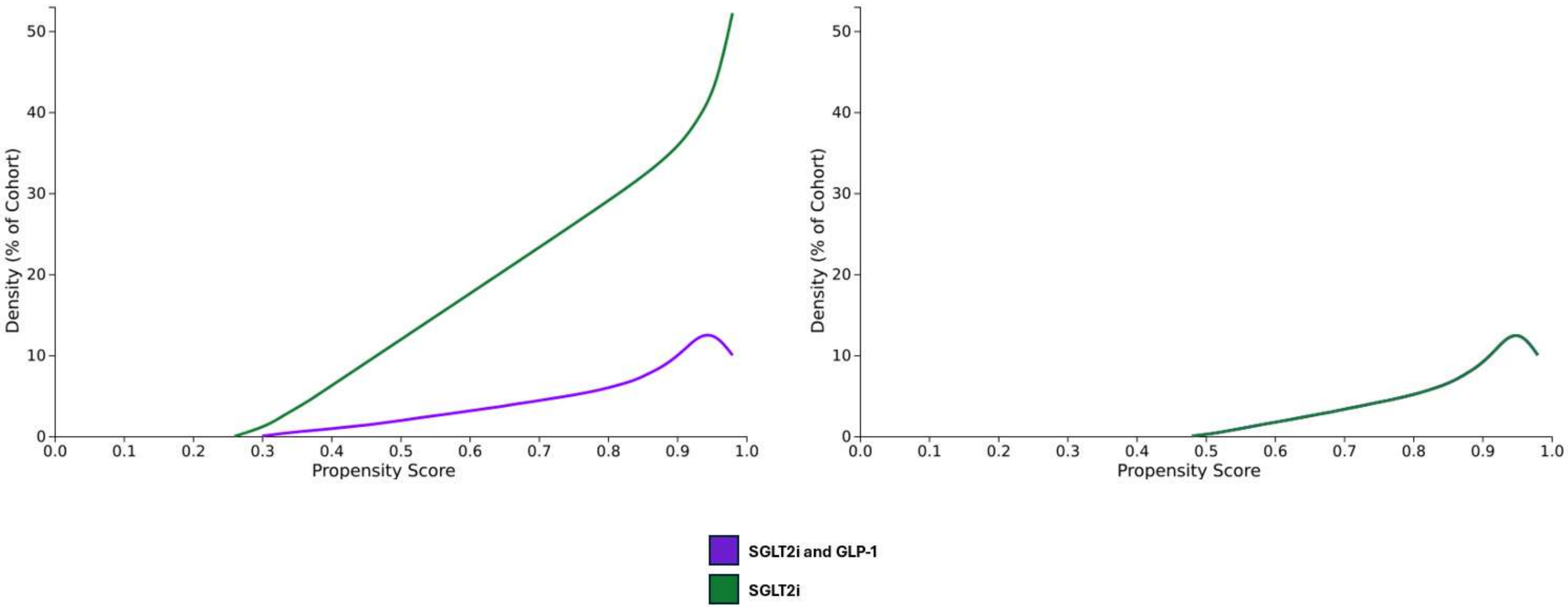

Supplementary Figure S2. Kaplan–Meier curves for All-cause Hospitalization at 1-Year

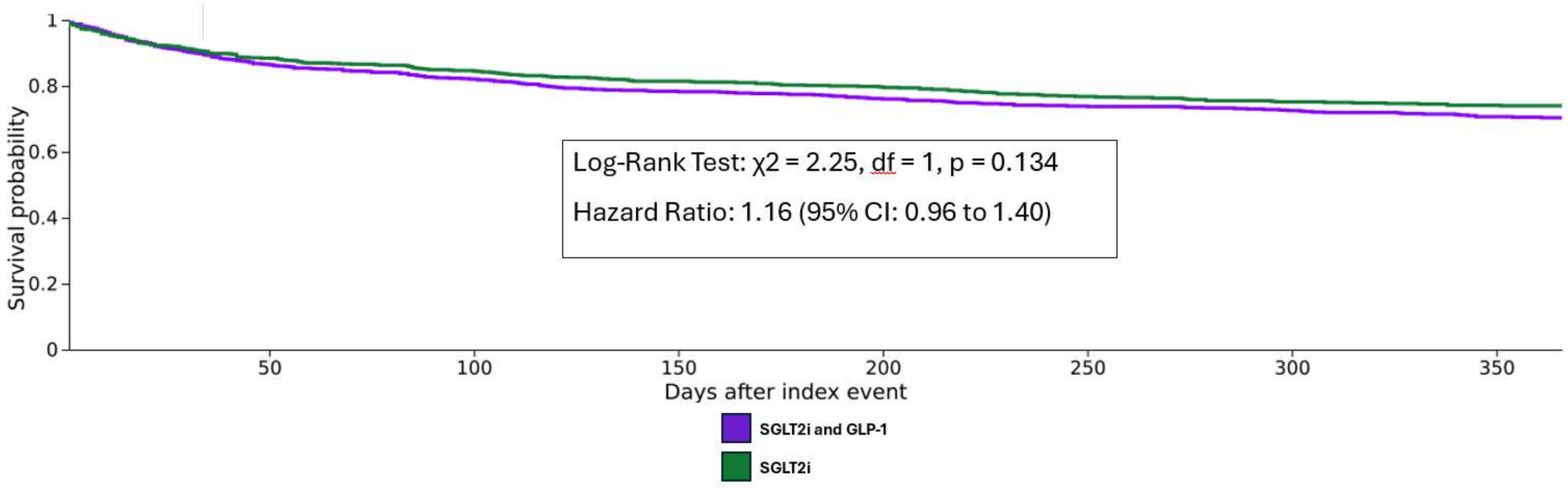

Supplementary Figure S3: Kaplan–Meier curves for Acute Myocardial Infarction at 1-Year

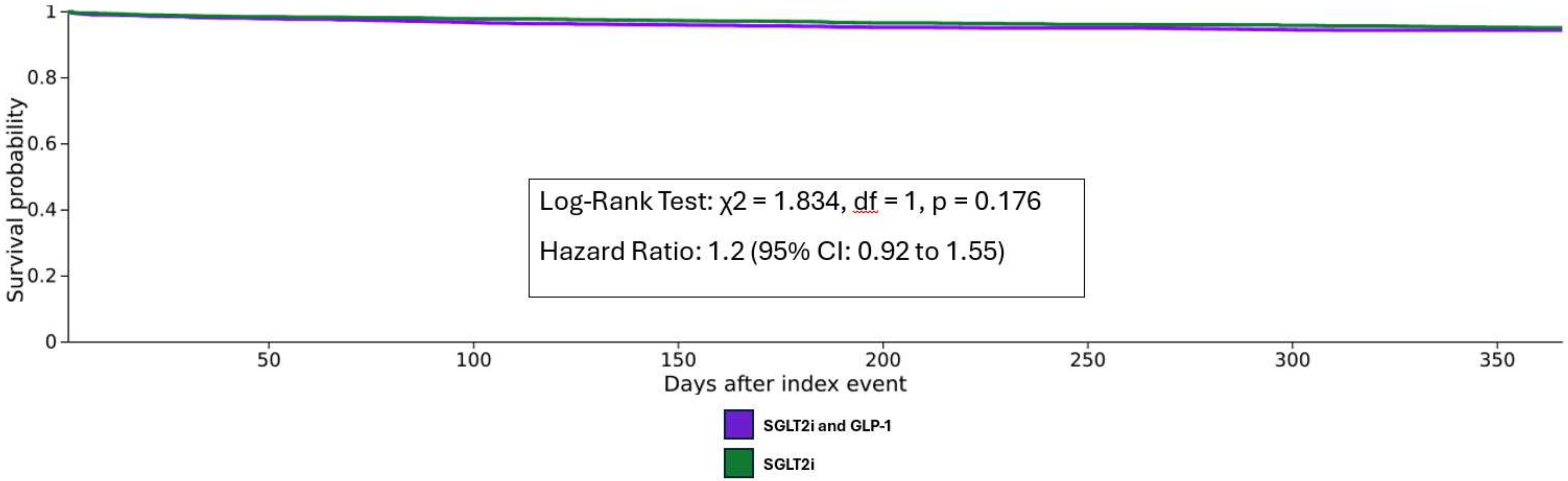

Supplementary Figure S4. Kaplan–Meier curves for Incident Atrial Fibrillation at 1-Year

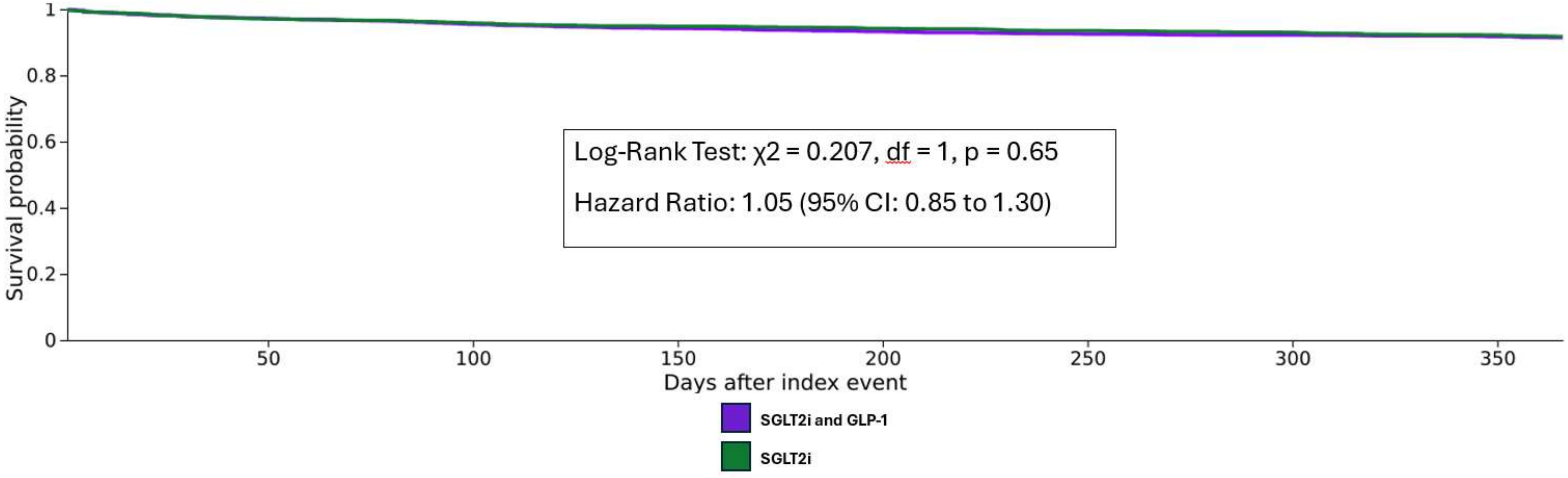

Supplementary Figure S5. Kaplan–Meier curves for Acute Kidney Failure at 1-Year

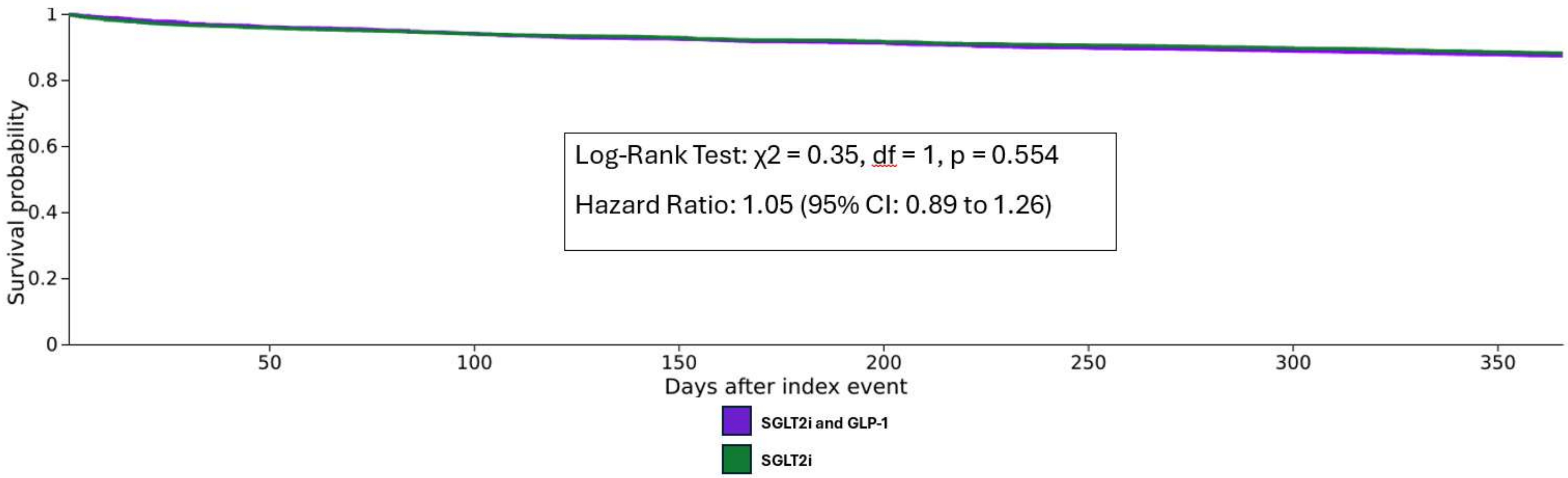

Supplementary Figure S6. Kaplan–Meier curves for Pulmonary Edema at 1-Year

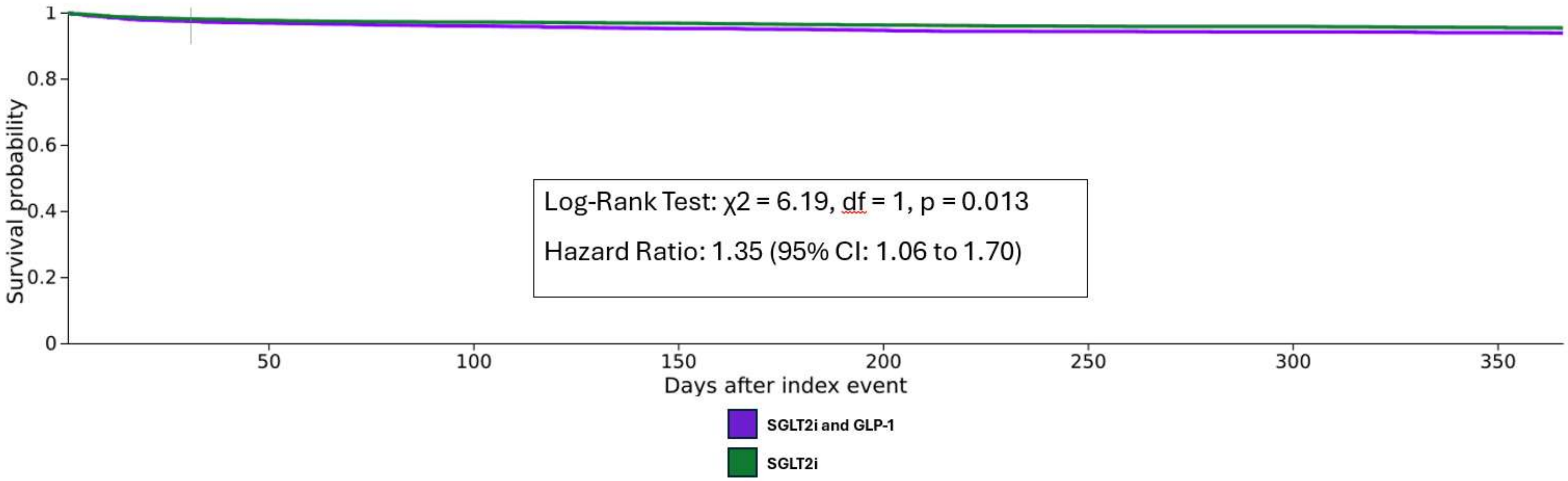

Supplementary Figure S7. Kaplan–Meier curves for New Onset Diuretics at 1-Year

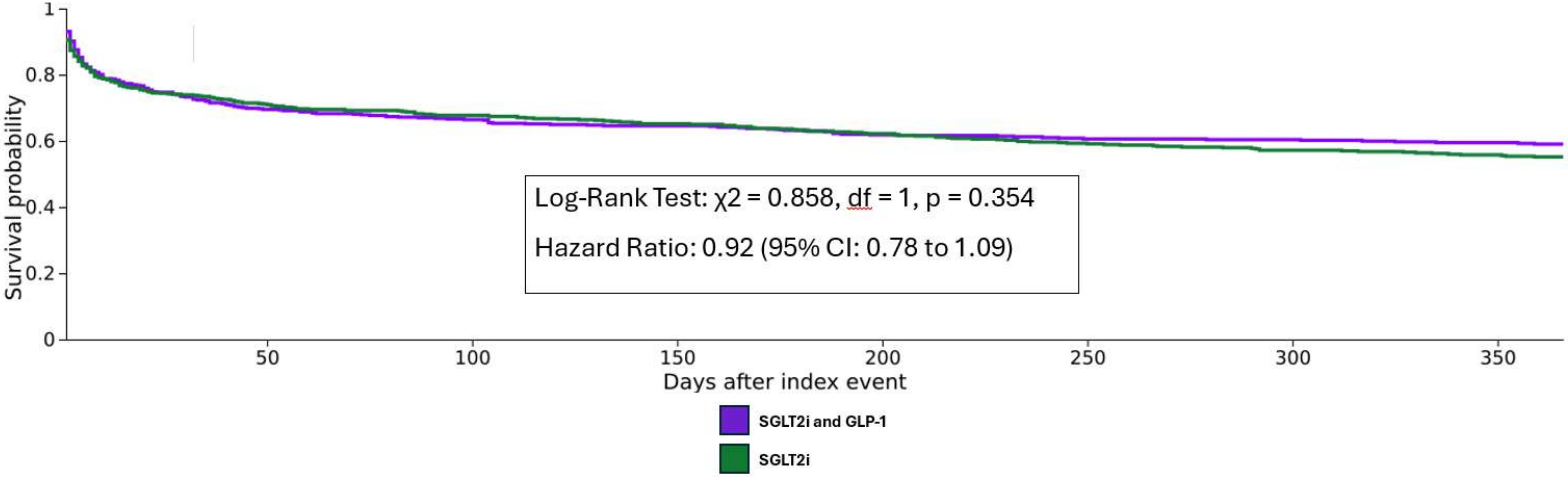

Supplementary Figure S8. Kaplan–Meier curves for Urinary Tract Infection at 1-Year

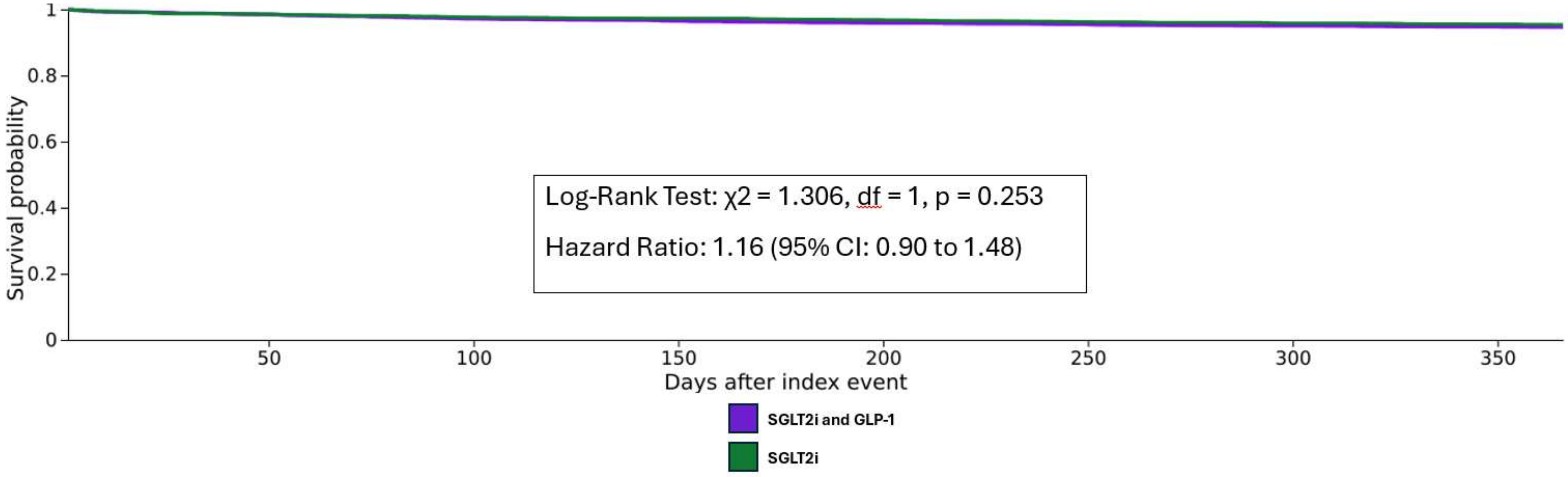

Supplementary Figure S9. Kaplan–Meier curves for Retinopathy at 1-Year

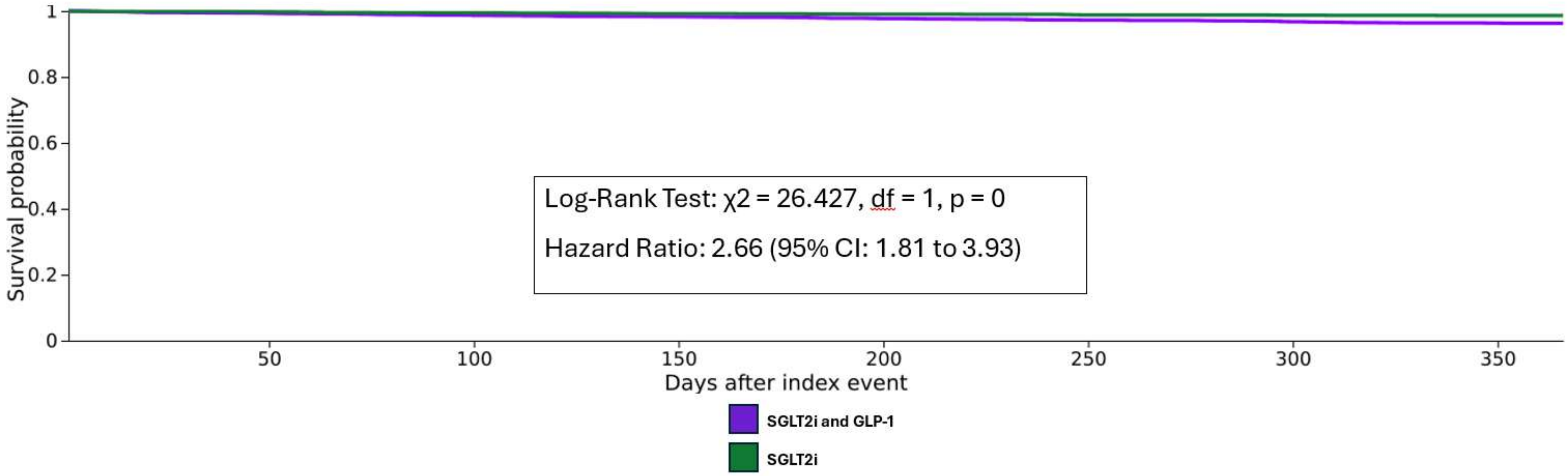

Supplementary Figure S10. Kaplan–Meier curves for Hypoglycemia at 1-Year

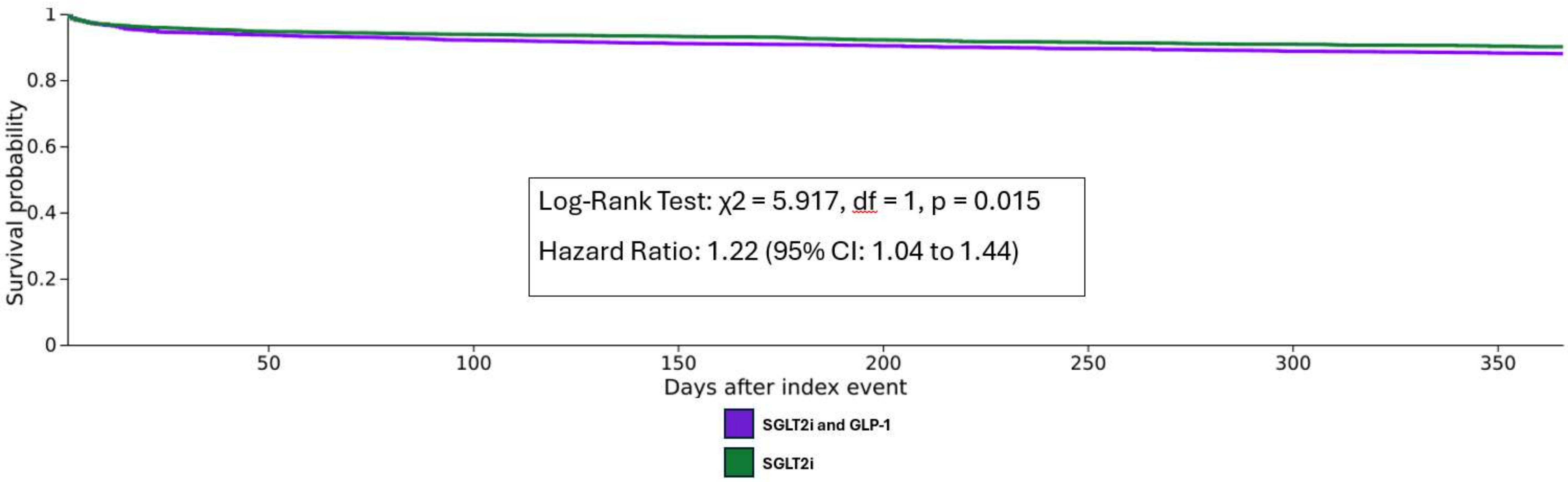

Supplement: Supplementary file 1 [file biomedicines-14-01368-s001.zip › biomedicines-4310583-supplementary.pdf]
